# *Bacillus subtilis* DisA regulates RecA-mediated DNA strand exchange

**DOI:** 10.1093/nar/gkz219

**Published:** 2019-03-27

**Authors:** Rubén Torres, Begoña Carrasco, Carolina Gándara, Amit K Baidya, Sigal Ben-Yehuda, Juan C Alonso

**Affiliations:** 1 Department of Microbial Biotechnology, Centro Nacional de Biotecnología, CNB-CSIC, 3 Darwin St, 28049 Madrid, Spain; 2 Department of Microbiology and Molecular Genetics, Institute for Medical Research Israel-Canada, The Hebrew University-Hadassah Medical School, POB 12272, The Hebrew University of Jerusalem, 91120 Jerusalem, Israel

## Abstract

*Bacillus subtilis* diadenylate cyclase DisA converts two ATPs into c-di-AMP, but this activity is suppressed upon interaction with sites of DNA damage. DisA forms a rapid moving focus that pauses upon induction of DNA damage during spore development. We report that DisA pausing, however, was not observed in the absence of the RecO mediator or of the RecA recombinase, suggesting that DisA binds to recombination intermediates formed by RecA in concert with RecO. DisA, which physically interacts with RecA, was found to reduce its ATPase activity without competing for nucleotides or ssDNA. Furthermore, increasing DisA concentrations inhibit RecA-mediated DNA strand exchange, but this inhibition failed to occur when RecA was added prior to DisA, and was independent of RecA-mediated nucleotide hydrolysis or increasing concentrations of c-di-AMP. We propose that DisA may preserve genome integrity by downregulating RecA activities at several steps of the DNA damage tolerance pathway, allowing time for the repair machineries to restore genome stability. DisA might reduce RecA-mediated template switching by binding to a stalled or reversed fork.

## INTRODUCTION

To combat threats posed by DNA damage, cells have evolved mechanisms—collectively termed the DNA-damage response—to detect DNA lesions, signal their presence and promote the recruitment of repair factors to the damaged site in a well-orchestrated manner (reviewed in 1, 2–4). In eukaryotic cells, sensor proteins recognize DNA lesions that cause replication fork stalling and accumulation of single-stranded (ss) DNA regions coated by the essential single-stranded binding protein RPA (termed SSB or SsbA in bacteria). RPA bound to the ssDNA recruits a checkpoint protein to the damaged region signalling an orchestrated and hierarchically coordinated cellular response to replicative stress (1,4). Other sensor proteins recognize DNA breaks and recruit a checkpoint protein at a double-strand break (DSB) (3,5–8). The activated checkpoint proteins then recruit accessory factors, implicated in the repair of RPA-coated ssDNA regions and DSBs (1–8).

In the presence of DNA damage, *Bacillus subtilis* fails to initiate the developmental process of sporulation (9). DisA, which is involved in monitoring genomic stability at the onset of sporulation, is a key signalling component that acts as a checkpoint to maintain genome integrity (10–13). DisA is organized into focal assemblies that move dynamically along the nucleoid, monitoring genome integrity (10). The switch from DisA-mediated recognition of its discrete target to the repair machinery requires controlled steps. DisA pauses at endonuclease (HO), nalidixic acid (Nal) or mitomycin C (MMC)-induced damage sites. The DisA-dependent DNA-damage checkpoint is activated, and then a cellular response that culminates in a temporary block in sporulation is triggered (10,14). DisA might regulate DNA repair by recruiting repair factors to the damage site, and by halting cellular development it increases the time available for DNA repair before replication ensues.

DisA is composed of an N-terminal globular domain featuring diadenylate cyclase (DAC) activity, and a C-terminal RuvA-like Holliday junction (HJ) DNA-binding domain, separated by a central helical domain (15). *In vitro, B. subtilis* DisA preferentially binds displacement loops (D-loops, also termed 3-way junctions) and HJ intermediates (also termed four-way junctions), and with lower affinity ssDNA and double-stranded (ds) DNA (12,15). DisA converts a pair of ATPs (or ADPs) into cyclic 3′,5′-adenosine monophosphate (c-di-AMP), but such DAC activity is suppressed when DisA binds a branched DNA structure (D-loops, HJs and stalled replication forks) (12,15). This crucial second messenger is synthesized by almost all bacteria, with the exception of Proteobacteria (16,17).

Inactivation of *disA* renders cells sensitive to damaged DNA template bases or DNA distortions that stall the replicative polymerase (11–13). If unrepaired, these DNA lesions at stalled replication forks result in discontinuities opposite the damage in the newly synthesized DNA strand. This replicative stress contributes to circumvent such arrest via RecA-mediated template switching or fork reversal or via the less characterized pathway of translesion synthesis (18–22). The *disA* gene is epistatic to *recA* in response to a replicative stress (12,13). It has been reported that *Escherichia coli* or *B. subtilis* RecA attenuate fork progression, and a failure to restart replication often leads to gross chromosomal rearrangements and cell death (23,24). Thus, a critical question is where, when and how RecA is regulated in concert with DisA. Before characterizing such a molecular interaction, it is also crucial to define what is DisA sensing (DSBs, SsbA-coated ssDNA regions, recombination intermediates), and if DisA or its product (c-di-AMP) evolved to coordinate RecA-mediated error-free DNA damage tolerance pathways.

RecA protein acts at several steps of the homologous recombination pathway for DNA DSB repair, gap-filling mechanisms and post-replication repair. Canonical DSB repair in *B. subtilis* cells is initiated by the recognition of the primary lesion by RecN, followed by a nucleolytic resection processed by the helicase-nuclease AddAB (counterpart of *E. coli* RecBCD or *Mycobacterium tuberculosis* AdnAB) or by the RecJ exonuclease in concert with a RecQ-like DNA helicase (RecQ and/or RecS) (25–28). Inactivation of *addAB* and *recJ* impairs cells in DSB repair to levels similar to the absence of RecA (29). End resection generates an overhanging 3′-ssDNA onto which SsbA binds, but it has to be outcompeted by RecA mediators for RecA to bind the ssDNA substrate (30–32). *In vitro, B. subtilis* RecA is a ssDNA-dependent ATPase and dATPase (33,34). Nucleotide binding is required for RecA nucleation and polymerization onto ssDNA, and a RecA nucleoprotein filament conducts all the catalytic steps of recombination in the presence of accessory proteins (35). Nucleotide hydrolysis throughout the filament contributes to cycles of ssDNA binding (RecA in the ATP or dATP bound form, RecA·(d)ATP) and dissociation (RecA·(d)ADP). The cooperative RecA·(d)ADP interaction among different monomers stabilizes the filament, thus dissociation of RecA·(d)ADP monomers occurs predominantly from the filament ends (31,32).

RecA·ATP cannot nucleate onto SsbA-coated ssDNA, but it can nucleate and polymerize on the RecO-ssDNA-SsbA complexes (35,36). Then, RecA·ATP, with the help of the two-component mediator (SsbA and RecO) is activated to catalyse DNA strand exchange *in vitro. In vivo*, RecA, with the help of mediators (RecO and RecR) and modulators (RecF, RecX, RecU) and using ATP as a cofactor, assembles onto SsbA-coated ssDNA (35,37,38). A RecA·ATP nucleoprotein filament promotes homology search in the intact sister-strand or sister-chromosome, followed by DNA strand invasion, formation of a D-loop and DNA strand exchange activities, that are largely independent of its ATPase activity (31,32). However, RecA·dATP, that binds ssDNA with high affinity and cooperativity, can nucleate on the SsbA–ssDNA complexes and catalyse DNA strand exchange even in the absence of accessory proteins (33,34,36).

In this study, using a combination of cytological and biochemical assays, we show that in response to unrepaired DNA damage, DisA pauses at RecA-mediated recombination intermediates. DisA interacts with and inhibits RecA filament growth and RecA-mediated DNA strand exchange. This effect is independent of nucleotide hydrolysis by RecA or increasing concentrations of the DisA product c-di-AMP. We propose that the DisA surveillance mechanism recognizes a D-loop or stalled fork at unrepaired DNA lesions, and negatively regulates RecA-mediated annealing of nascent DNA strands to initiate DNA synthesis from an alternative template (template switching and fork reversal). In exponentially growing cells, DisA enables flexibility in replication fork repair without committing repair via DNA breaks, and reduces cell proliferation by lowering c-di-AMP levels.

## MATERIALS AND METHODS

### Bacterial strain and plasmids

*E. coli* BL21(DE3) cells bearing pLysS and compatible plasmids were utilized for protein overexpression, XL1-Blue cells for plasmid amplification and BTH101 cells bearing plasmids pUT18 and pUT18C (to generate fusions at the N- and C-termini of the T18 domain, respectively), pKNT25 and pKT25 (to generate fusions at the N- and C-termini of the T25 domain, respectively) derivatives were used for bacterial two-hybrid analysis. Plasmids pKT25-zip and pUT18C-zip were used as controls. *B. subtilis* BG214 and its isogeneic derivatives are described (Supplementary Table S1) (39,40). By site-directed mutagenesis codon 77 (GAT) was replaced by AAT rendering a *disA* D77N active site mutant. The DNA of wild-type (*wt*) *disA, disA* ΔC290 or *disA* D77N gene under the control of an IPTG-inducible promoter was used to transform competent *B. subtilis* BG214 (*rec*^+^) or BG1245 (*ΔdisA*) cells, and thus it was integrated into the *amyE* locus with selection for spectinomycin resistance (Spc^R^) (Supplementary Table S1).

The *disA-gfp* gene, which is expressed from its native locus and promoter, shows a *wt* phenotype when exposed to DNA damaging agents, as described (10). The *disA-gfp* gene was transferred to BG214 and its isogenic derivatives listed in Supplementary Table S1 by SPP1-mediated phage transduction. The Δ*recA* mutation was moved into the *disA-gfp* strain by SPP1-mediated phage transduction (Supplementary Table S1). The 3′-end of the *disA* ΔC290 gene was fused to the *gfp* gene to render plasmid-borne *disA* ΔC290-*gfp* gene. Plasmid-borne *disA* ΔC290-*gfp* DNA was used to transform the BG214 strain; integration occurs into its native locus and expression is driven by its native promoter (see Supplementary Table S1).

*E. coli* cells bearing pCB722-*ssbA*, pCB669-*recO*, pCB875-*disA*, pCB1081-*disA*ΔC290 and pCB1080-borne *disA* D77N gene were used to over-express the *ssbA, recO, disA, disA* ΔC290 or *disA* D77N gene, respectively (11,41–43). *B. subtilis* BG214 cells bearing pBT61 were used to over-express the *recA* gene (44). The 3,199-bp pGEM3 Zf(+) was used as a source of ssDNA and dsDNA (Promega Biotech, Spain).

### Fluorescence microscopy

Growth of *B. subtilis* was carried out in hydrolysed casein (CH) growth medium. The cultures were inoculated at an OD_600_ of 0.05 from an overnight culture in the same medium (10). Sporulation was induced at 32°C by transferring cells growing in CH medium to the resuspension medium of Sterlini and Mandelstam (10,45). *B. subtilis* BG1733 (*disA-gfp*), BG1743 (*disA-gfp* Δ*addAB* Δ*recJ*), BG1741 (*disA-gfp* Δ*recO*), BG1737 (*disA-gfp* Δ*recA*) and BG1745 (*disA* ΔC290*-gfp*) cells were treated with different concentrations of Nal (targets DNA gyrase and prevents religation of DNA strands broken by DNA gyrase activity) or MMC (a crosslinking agent) at the onset of sporulation.

Fluorescence microscopy was carried out as previously described (46). For time-lapse microscopy observations, a chamber filled with sporulation medium containing 1.5% agarose was used. Samples (0.5 ml) of culture were removed, centrifuged, and resuspended in 10 μl of PBS × 1 (Phosphate-Buffered Saline) supplemented with the membrane stain FM 4–64 (Molecular Probes, Eugene, OR, USA) at 1 μg/ml and the DNA stain 4,6-diamidino-2-phenylindole (DAPI) (Sigma) at 2 μg/ml and incubated in a temperature-controlled chamber at 32°C. Importantly, DisA movement was not affected by the presence of DAPI. Cells were visualized and photographed using Axio Observer Z1 (Zeiss) or Eclipse Ti2 (Nikon) microscopes equipped with CoolSnap HQ camera (Photometrics, Roper Scientific). System control, image processing and foci speed measurement were performed using MetaMorph 6.2r4 software (Universal Imaging) and NIS-Elements Advanced Research (Nikon). Foci speed was determined by imaging cells with 400 ms stream acquisition, upon tracking the position of each individual protein molecule over a 60 s interval. Using particle tracking tools, the position for each fluorophore at each time point was determined. The distance the particle has moved in a given time period allow us to infer the speed of the focus. Foci speed is shown as the mean of the speeds of each individual focus ± SEM.

### Reagents, DNA and protein purification

All chemicals used in this study were of analytical grade. Isopropyl β-d-1-thiogalactopyranoside (IPTG) was from Calbiochem and polyethyleneimine (PEI), DTT, c-di-AMP, ATP, dATP and ATPγS were from Sigma-Aldrich. DNA restriction enzymes, T4 polynucleotide kinase and DNA ligase were supplied by New England Biolabs. DEAE, Q- and SP-Sepharose were from GE healthcare, hydroxyapatite from BioRad, phosphocellulose was from Whatman and the Ni-column from Qiagen. The radioactive nucleotide [α-^32^P]-ATP was from Perkin Elmer.

CsCl purified pGEM3 Zf(+) plasmid dsDNA and circular pGEM3 Zf(+) ssDNA were used as recombination substrates. If not stated otherwise, DNA quantities are expressed as mols of nucleotides (nt).

*E. coli* BL21(DE3) [pLysS] cells bearing pCB722-*ssbA*, pCB669-*recO* or pCB875-borne *disA* gene were used to purify SsbA, RecO or DisA as described (11,36,41). *E. coli* BL21(DE3) [pLysS] cells bearing pCB1081-*disA* ΔC290 or pCB1080-borne *disA* D77N gene were used to purify DisA ΔC290 or DisA D77N. *B. subtilis* BG214 cells bearing pBT61-borne *recA* were used to over-express and purify RecA as reported (40).

The purified SsbA (18.7 kDa), RecO (29.3 kDa), DisA (40.7 kDa), DisA ΔC290 (33.5 kDa), DisA D77N (40.7 kDa) and RecA (38.0 kDa) proteins were >95% pure based on staining after SDS-PAGE and identified by degradation and MALDI-TOF/TOF analysis. The DisA protein (apparent molecular mass 40 kDa) co-purified with a 41 kDa polypeptide. Protein identification by peptide mass finger printing analysis revealed that both proteins were DisA and DisA-bound c-di-AMP. The molar extinction coefficients for SsbA, RecO, DisA and RecA were calculated as 11,400; 19,600; 22,350 and 15,200 M^−1^ cm^−1^ at 280 nm as previously described (40). The SsbA, RecO, DisA and RecA concentrations were determined using the aforementioned molar extinction coefficients. RecA and DisA are expressed as moles of monomeric, RecO as dimeric and SsbA as tetrameric proteins. In exponentially growing unperturbed *wt* cells, the physiological concentrations of c-di-AMP (∼3 μM) and RecA (∼5.5 μM as monomers) are similar, but significantly higher than those of DisA (∼0.8 μM as monomers) (11,12,47).

### Protein-protein interaction

*In vivo* protein–protein interaction was assayed using the adenylate cyclase-based bacterial two-hybrid technique as described (48). The plasmid-borne T18 or T25 catalytic domain of the *Bordetella* adenylate cyclase gene was fused to the 5′- and 3′-end of the *disA* and *recA* genes, to render fusions at the N- and C-termini of the resulting proteins to be tested. After co-transformation of the plasmid pairs producing the complementary fusion proteins into the reporter BTH101 strain, different dilutions were spotted onto LB plates supplemented with ampicillin, kanamycin, streptomycin, 1 mM IPTG and 10% X-Gal. The plates were then incubated at 25°C for 3–4 days. The empty vectors (negative control) or the pKT25-zip and pUT18C-zip vectors (positive control) were co-transformed into the reporter strain and spotted onto the screening medium plate. Each transformation was done at least in triplicate and a representative result is shown.

*In vitro* protein–protein interaction was assayed using the His-tagged DisA alone (1.5 μg), RecA (1.5 μg) or His-DisA mixed with RecA (1.5 μg each) in the presence or the absence of ssDNA and ATP. Proteins were loaded onto a 50-μl Ni^2+^ microcolumn at room temperature in Buffer A (50 mM Tris–HCl pH 7.5, 50 mM NaCl, 10 mM MgCl_2_, 5% glycerol) containing 20 mM imidazole. When indicated, buffer A contained 5 mM ATP and 10 μM ssDNA. After extensive washing, the retained proteins were eluted with 50-μl of Buffer A containing 1 M NaCl and 400 mM imidazole. The proteins were separated by 17.5% SDS-PAGE, stained with Coomassie Blue and detected using anti-His-tag monoclonal and anti-RecA-polyclonal antibodies by Western blot.

### Analysis of c-di-AMP production and ATPase assays *in vitro*

The DAC activity of DisA was studied by monitoring the formation of c-di-AMP using thin-layer chromatography (TLC) and [α-^32^P]-ATP with a modified protocol (12,15). DAC reactions were performed at 37°C using a range of protein concentrations or variable times, in buffer B (50 mM Tris–HCl (pH 7.5), 1 mM DTT, 80 mM NaCl, 10 mM MgOAc [magnesium acetate], 50 μg/ml BSA, 5% glycerol) containing increasing ATP or dATP concentrations (0.1, 1 and 5 mM) (at a ratio of 1:2,000, 1:20,000 and 1:100,000 [α^32^P]-ATP:(d)ATP, respectively). The reaction was stopped by adding 25 mM EDTA. From each reaction, 2–5 μl were spotted in 20 × 20 cm TLC PEI cellulose plates, and run for ∼2 h in a TLC chamber containing running buffer C (1:1 [v/v] 1.5 M KH_2_PO_4_ [pH 3.6] and 70% ammonium sulfate). Dried TLC plates were analysed by phosphor imaging. Spots were quantified using ImageJ (NIH).

The ssDNA-dependent ATP/dATP (denoted as [d]ATP) hydrolysis activity of RecA protein was assayed via a (d)ATP/NADH-coupled spectrophotometric enzyme assay as described (42,49). Assays were done in buffer B containing 5 mM ATP or dATP for 30 min at 37°C, as described (42). When indicated, increasing concentrations of c-di-AMP were added to the reaction.

The orders of addition of 3,199-nt pGEM3 Zf(+) ssDNA (10 μM in nt), 3,199-bp pGEM3Zf (+) *Kpn*I-linearized dsDNA (20 μM in nt) and the purified proteins are indicated in the text. The data obtained from (d)ATP hydrolysis were converted to (d)ADP and plotted as a function of time as described (42). As reported (42,50), the rate of (d)ATP hydrolysis was derived from the slope of the linear part of the curves. The lag time, which represents the delay in reaction progress relative to a theoretical reaction curve lacking it, was derived from the time intercept of a linear regression line fit to the steady state portion of data in (d)ATP hydrolysis assays, as reported (42,50).

### RecA-mediated DNA strand exchange

Standard reactions containing 3,199-bp *Kpn*I-cleaved pGEM3 Zf(+) dsDNA (20 μM in nt) and the homologous circular 3,199-nt ssDNA (10 μM in nt) were pre-incubated with the indicated protein or protein combination in buffer B containing 5 mM dATP, ATP or ATPγS for 5 min at 37°C. Then, a fixed RecA or a variable DisA concentration was added and the reaction was incubated for fixed or variable times at 37°C. A (d)ATP regeneration system (8 units/ml creatine phosphokinase and 8 mM phosphocreatine) was included in the recombination reaction. After incubation of the recombination reaction, samples were de-proteinised as described (51) and fractionated through 0.8% agarose gel electrophoresis with ethidium bromide. The signal of the remaining DNA substrate (*lds*), and the appearance of intermediates (*jm*) and products (*nc*) were quantified from the gels using a Gel Doc (BioRad) system, as described (41). When indicated, the sum of *jm* and *nc* are shown (% of recombination).

## RESULTS

### End resection is dispensable for DisA pausing after DNA damage

**Figure 1.**
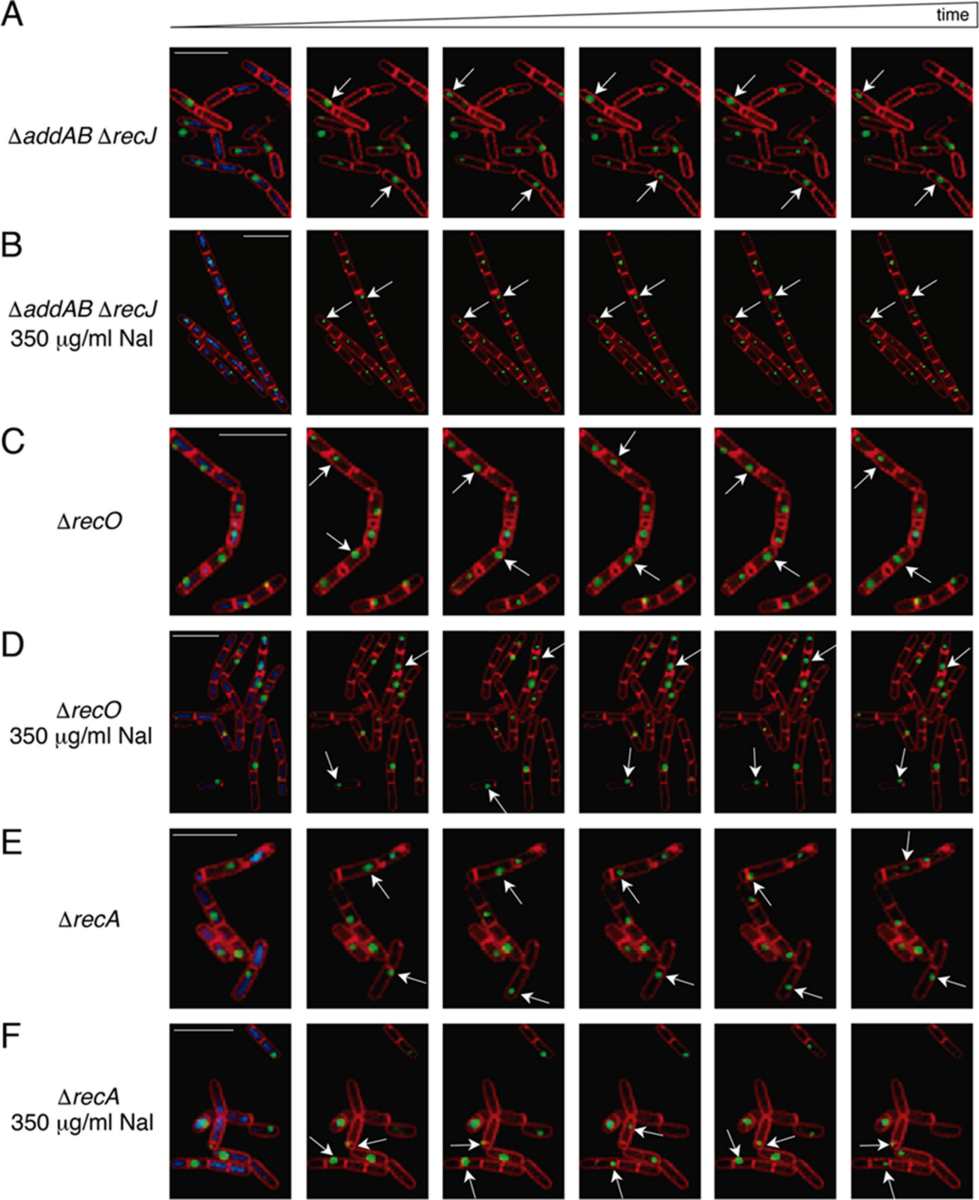
DisA foci remain highly dynamic upon DNA damage in Δ*recO* or Δ*recA* cells. (**A**–**F**) Dynamic localization of DisA-GFP foci demonstrated by time-lapse microscopy from individual cells of the DisA-GFP-producing strain. Time-lapse microscopy images (400 ms intervals) from DisA-GFP-producing strains stained with DAPI (blue), FM 4–64 (red) and DisA-GFP (green) seeing as foci in unperturbed Δ*addAB* Δ*recJ* (A), Δ*recO* (C) or Δ*recA* cells (E). The corresponding movies are displayed in the Supplemental data (Movies S3, S5 and S7). Time-lapse microscopy images (400 ms intervals) from DisA-GFP-producing strains in Δ*addAB* Δ*recJ* (B), Δ*recO* (D) or Δ*recA* cells (F) upon addition of 350 μg/ml Nal at the onset of sporulation. The corresponding movies are displayed in the Supplemental data (Movies S4, S6 and S8). Selected images were chosen for demonstration. Scale bars correspond to 5 μm.

To identify the type of lesions sensed by DisA *in vivo*, key proteins that process the DNA end (AddAB and RecJ), load RecA onto SsbA-coated ssDNA (RecO) or search for homology, mediate DNA strand invasion and catalyse DNA strand exchange (RecA) were inactivated in cells bearing a DisA-GFP fusion, and pausing of DisA in the presence of DNA damage was investigated. As previously documented (10), at the onset of sporulation DisA forms a discrete and dynamic focus that pauses upon sensing DNA damage produced by Nal (Supplementary Figure S1, movies S1 and S2, Table 1). As earlier described (10), the speed of movement of these foci was highly variable due to the scanning activity of DisA. To examine whether DisA pausing is dependent on processing of the DNA ends, we studied DisA-GFP dynamics in the Δ*addAB* Δ*recJ* cells. In unperturbed sporulating Δ*addAB* Δ*recJ* cells, DisA-GFP forms a discrete highly mobile focus that presumably associates with the nucleoid (Figure 1A, movie S3). As in *wt* unperturbed cells (Supplementary Figure S1, movie S1, Table 1) (10), ∼43% of the Δ*addAB* Δ*recJ* cells displayed a dynamic DisA-GFP focus as early as 20 min following sporulation induction, reaching ∼94% of the population at 120 min (Figure 1A, movie S3). In the absence of DNA damage, the DisA-GFP focus speed was estimated as 0.25 ± 0.09 μm s^−1^ in both *wt* and Δ*addAB* Δ*recJ* cells (Table 1). Similarly to *wt*, in the presence of Nal-induced lesions, the movement of DisA-GFP was halted in the majority of Δ*addAB* Δ*recJ* cells (Figure 1B, movie S4), with a measured speed of 0.02 ± 0.02 μm s^−1^ (Table 1). These results suggest that the end resection activity of AddAB and RecJ is not required for DisA stalling on the damage site.

**Table 1.** DisA diffusion rate

Strain	Nal (μg/ml)	Cells analysed^a^	Cells with focus (%)	Mean focus speed^b^ (μm s^−1^)
*rec* ^+^	0	1097	1044 (95.2)	0.24 ± 0.1
	350	973	934 (96)	0.04 ± 0.03
Δ*addAB* Δ*recJ*	0	1108	1033 (93.3)	0.25 ± 0.09
	350	931	877 (94.2)	0.02 ± 0.02
Δ*recA*	0	1116	1065 (95.5)	0.25 ± 0.1
	350	1087	1040 (95.7)	0.26 ± 0.1
Δ*recO*	0	1077	1039 (96.5)	0.25 ± 0.1
	350	1063	1012 (95.2)	0.25 ± 0.09
*disA* ΔC290-*gfp*	0	939	887 (94.5)	0.23 ± 0.08
	350	954	887 (93)	0.24 ± 0.09

^a^Total number of cells analyzed.

^b^The mean speed rate (μm s^−1^) ± SEM was calculated from >4 independent experiments.

### RecO and RecA are required for DisA pausing at sites of DNA damage

Next, we tested whether the signal recognized by DisA is generated before RecO outcompetes the high affinity binding of SsbA to ssDNA, and recruits RecA onto SsbA-coated ssDNA, or after RecA filament growth and RecA-mediated DNA strand invasion. To this end, DisA-GFP scanning along the chromosomes was investigated in the absence of the RecO mediator or the RecA recombinase. In the absence of Nal-induced lesions, DisA-GFP foci remained highly mobile in sporulating Δ*recO* or Δ*recA* cells (Figure 1C and 1E, movies S5 and S7) with *in bulk* mean speed rates of 0.25 ± 0.1 μm s^−1^ (Table 1), suggesting that absence of RecO or RecA does not affect the scanning activity of DisA. Intriguingly, DisA-GFP foci failed to stall and remained highly mobile in the presence of Nal-induced lesions in both sporulating Δ*recO* (Figure 1D, movie S6) or Δ*recA* cells (Figure 1F, movie S8). Further, the mean speed rates of 0.25 ± 0.09 and 0.26 ± 0.1 μm s^−1^ (Table 1) were similar to DisA dynamics in non-treated sporulating cells. These results indicate that DisA recognizes intermediate DNA structures that accumulate at or downstream of RecO and RecA activities. It is likely that DisA-GFP senses RecA nucleoprotein filaments or RecA-mediated recombination intermediates; such as D-loops or reversed stalled forks (HJ-like structure), produced by RecA with the help of the RecO mediator.

### DisA binding to DNA is required for pausing at a damage site

To test whether DisA activity involves frequent binding to and dissociation from DNA, and if the cease of DisA movement primarily requires the recognition of a cognate DNA substrate, we deleted the DisA HhH DNA binding domain, rendering DisA ΔC290 mutant. The *disA* ΔC290 gene was fused to *gfp*, and the construct was used to replace the endogenous *disA* gene. The *disA* ΔC290-*gfp* strain was subjected to sporulation followed by fluorescent microscopy. In the absence of DNA damage, the DisA ΔC290-GFP protein formed a focus that moved mainly in DNA free regions (Supplementary Figure S2A and S2C, Movie S9, Table 1). In the presence of Nal-induced DNA lesions, the majority of DisA ΔC290-GFP foci assembled outside the nucleoid and moved at a speed similar to that in the absence of DNA damage (Supplementary Figure S2B and D, Movies S9 versus S10, Table 1), suggesting that binding to DNA is required for DisA scanning and pausing at the DNA damage site.

To test whether deletion of the HhH motif (DisA ΔC290) affects the capacity to produce c-di-AMP, the protein was purified and analysed. As expected, *wt* DisA converted a pair of ATPs into c-di-AMP, but DisA D77N, carrying a D77N active site mutation that inactivates the DAC activity, did not (Supplementary Figure S2E) (12,15). The absence of HhH DNA binding domain reduced the DAC activity of DisA ΔC290 by 15- to 20-fold when compared to wt DisA (Supplementary Figure S2E), suggesting that *in vitro* the C-terminal DNA binding domain of DisA is crucial for its DAC activity. We then tested whether a *disA* ΔC290 mutation impairs cell survival (Supplementary Figure S2F). As revealed in Supplementary Material, *Annex 1*, in the absence of DNA damage ectopic expression of the *disA* ΔC290 mutant gene significantly reduced cell viability when compared to Δ*disA* or *disA* D77N cells (Supplementary Figure S2F). Deletion of *disA* or the *disA* D77N mutation decreased cell survival to methyl methanesulfonate (MMS) or the UV mimetic 4-nitroquinoline-1-oxide (4NQO) treatments. Expression of the *disA* ΔC290 mutant gene strongly reduced survival upon MMS or 4NQO treatment, indicating that the allele produces a dominant negative effect when compared to Δ*disA* or *disA* D77N cells (Supplementary Figure S2F).

### DisA interacts with RecA

**Figure 2.**
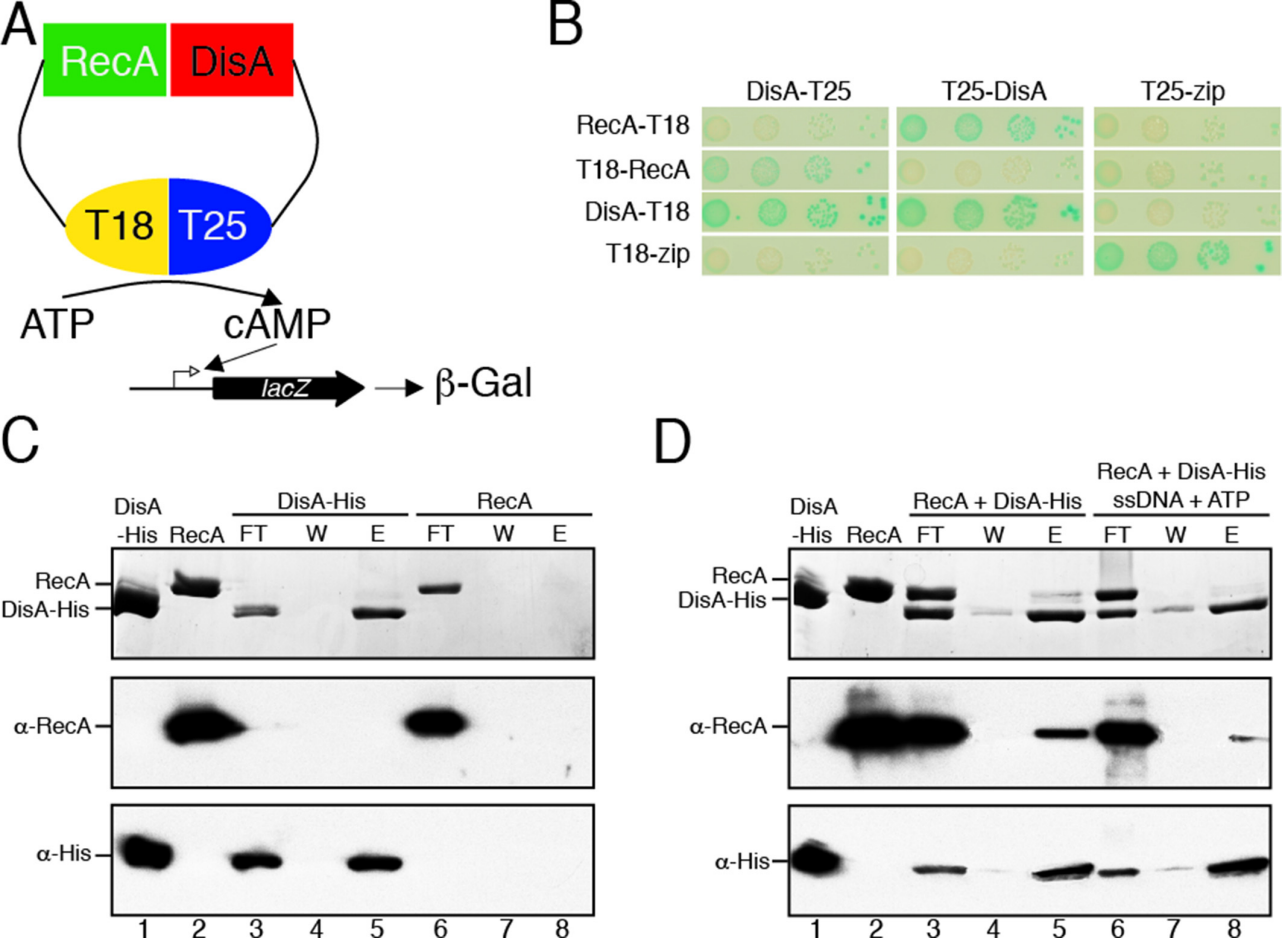
DisA interaction with RecA. (**A**) Scheme of the bacterial two-hybrid assay to test DisA-RecA *in vivo* interaction. (**B**) Bacterial two-hybrid assay shows an interaction of DisA with RecA *in vivo*. (**C**) His-tagged DisA alone (1.5 μg, lane 1) or native RecA alone (1.5 μg, lane 2) were loaded onto a 50-μl Ni^2+^ microcolumn at room temperature in Buffer A containing 20 mM imidazole. After extensive washing, the retained protein was eluted with 50-μl Buffer A containing 1 M NaCl and 0.4 M imidazole. (**D**) His-tagged DisA and native RecA (1.5 μg each) were loaded onto a 50-μl Ni^2+^ microcolumn at room temperature in Buffer A containing 20 mM imidazole in the presence or the absence of 5 mM ATP and 10 μM ssDNA. After extensive washing, the retained protein(s) was eluted with 50-μl Buffer A containing 1 M NaCl and 0.4 M imidazole. (C and D) The proteins present in the different fractions were separated by SDS-PAGE, and stained with Coomassie Blue. Western blot analysis of the protein mixtures highlighted with polyclonal anti-RecA (α-RecA) or monoclonal anti-His (α-His) antibodies was perfomed. FL, flow-through; W wash; E, elution with imidazole.The proteins present in the different fractions are indicated.

So far, we revealed that DisA binding to DNA is necessary for pausing at the damage site, and that RecA is required for DisA pausing. We therefore tested whether DisA physically interacts with RecA and/or with RecA-bound to ssDNA and ATP. To elucidate that, two different approaches were undertaken (see Supplementary Material, *Annex 2*). First, using a bacterial two-hybrid assay (Figure 2A), we confirmed that DisA interacts with itself and with RecA (Figure 2B). Second, His-tagged DisA was bound to a Ni^2+^ agarose column through coordination with the C-terminal histidine tag, in the absence or presence of ssDNA and ATP·Mg^2+^, but RecA alone was not retained by the Ni^2+^ matrix. However, in the presence of DisA, retention of apo RecA or RecA·ATP bound to ssDNA in the Ni^2+^ matrix was observed (see Supplementary Material, *Annex 2*, Figure 2C and D). These results indicate the direct physical interaction between these two proteins.

### DisA interferes RecA·ATP nucleation onto ssDNA

DisA interacts with RecA even in the apo form (Figure 2), and RecA is required for DisA pausing at the damage sites (Figure 1). To understand whether DisA regulates RecA nucleation and/or filament growth onto ssDNA, kinetics studies of RecA-mediated hydrolysis of ATP (or dATP), as an indirect readout of RecA binding to ssDNA, were used (31,32).

**Figure 3.**
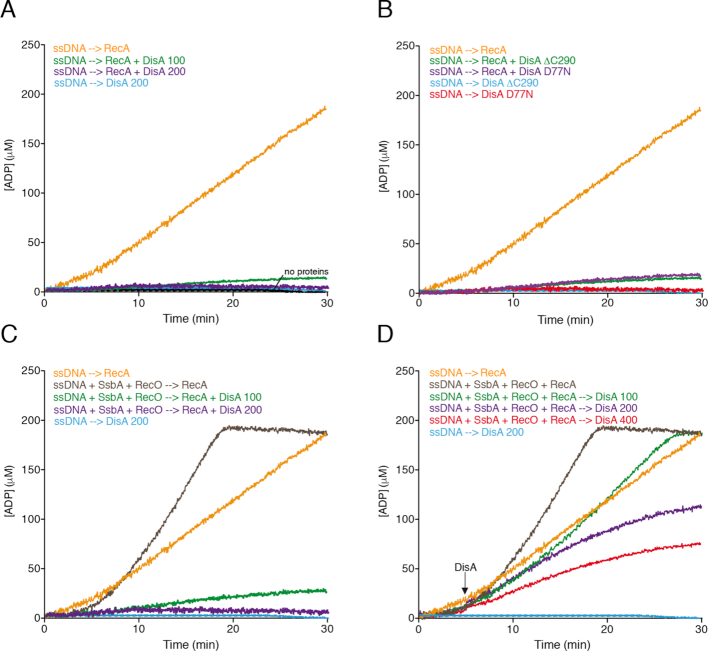
RecA-mediated ATPase activity in the presence of DisA. (**A**) Circular 3,199-nt ssDNA (10 μM in nt) was incubated with RecA (0.8 μM), DisA (0.1 or 0.2 μM) or both in buffer B containing 5 mM ATP and the ATPase activity measured for 30 min. (**B**) Circular ssDNA was incubated with RecA, DisA D77N or DisA ΔC290 (0.1 μM), or both in buffer B containing 5 mM ATP and the ATPase activity measured for 30 min. (**C**) Circular ssDNA was pre-incubated with stoichiometric SsbA (0.3 μM, 1 SsbA tetramer/33-nt) and RecO (0.2 μM, 1 RecO monomer/50-nt), and then incubated with RecA (0.8 μM, 1 RecA monomer/12-nt)), DisA (0.1 or 0.2 μM, 1 DisA monomer/100- and 50-nt) or both in buffer B containing 5 mM ATP and the ATPase activity measured for 30 min. (**D**) Circular ssDNA was pre-incubated with SsbA (0.3 μM) and RecO (0.2 μM), and then incubated with RecA (0.8 μM) in buffer B containing 5 mM ATP and the ATPase activity measured for 5 min. Then, DisA (0.1, 0.2 or 0.4 μM) was added and the ATPase activity measured for 25 min more. All reactions were repeated three or more times with similar results.

RecA hydrolyzed ATP at near the previously observed *K*_cat_ of 8.9 ± 0.3 min^−1^ (Figure 3A orange line, Supplementary Table S2) (35,36,41). In the presence of increasing sub-stoichiometric DisA concentrations, RecA-mediated ATP hydrolysis was inhibited at low DisA concentrations, and blocked at higher concentrations (Figure 3A, green and purple vs orange lines). This inhibition neither correlated with DisA-mediated hindering of the ssDNA substrate nor with any contamination (e.g. a protease) in the DisA preparation, as when RecA was replaced by its paralogous protein, RadA/Sms, the ATPase activity of the latter was not affected by DisA (Supplementary Figure S3A). As described in Supplementary Material, *Annex 3*, this inhibition does not correlate with the exhaustion of the nucleotide pool (Supplementary Figure S4).

To examine whether DisA or DisA-mediated c-di-AMP synthesis inhibits the ATPase activity of RecA, we have used DisA D77N that does not synthesize c-di-AMP (Supplementary Figure S2C). When DisA was replaced by DisA D77N, RecA-mediated ATP hydrolysis was blocked to a comparable level (Figure 3A versus B purple lines), showing that the inhibition of RecA-mediated ATP hydrolysis is independent of c-di-AMP synthesis. To corroborate this observation, RecA-mediated ATP hydrolysis in the presence of increasing c-di-AMP concentrations was measured. Saturating (1:125 RecA:c-di-AMP molar ratio) to oversaturating c-di-AMP concentrations (1:6,250 RecA:c-di-AMP molar ratio) did not affect the maximal rate of RecA-mediated ATP hydrolysis (Supplementary Figure S3B).

To address whether DisA competes with RecA for ssDNA binding, DisA was replaced by DisA ΔC290. DisA deleted of its C-terminal DNA binding domain also inhibited RecA-mediated ATP hydrolysis when compared to RecA alone (Figure 3B, green line). It is likely that DisA interacts with and inhibits the ATPase activity of RecA rather than c-di-AMP or a competition for ssDNA binding.

### DisA interferes RecA·ATP nucleation onto SsbA–ssDNA-RecO complexes

ssDNA is bound and protected by SsbA *in vivo*, and *B. subtilis* RecA·ATP cannot nucleate onto ssDNA-SsbA complexes, but it can nucleate and polymerize in the RecO–ssDNA–SsbA complexes more efficiently than on RecO-ssDNA complexes (35,36,52). To test whether a RecO-ssDNA-SsbA complex can suppress the negative effect exerted by DisA in RecA-mediated ATP hydrolysis, the ssDNA was pre-incubated with stoichiometric SsbA and RecO concentrations for 5 min. RecA and increasing DisA concentrations (1 DisA/100- to 50-nt) were added, and the ATP hydrolysis rate was measured (Figure 3C). In the presence of preformed RecO–ssDNA–SsbA complexes, ATPase activity of RecA showed a biphasic shape curve with ∼5 min lag phase, and the final steady state rate of ATP hydrolysis was significantly increased (*K*_cat_ of 18.0 ± 0.2 min^−1^), when compared to the absence of SsbA and RecO (*K*_cat_ of 8.9 ± 0.3 min^−1^) (Figure 3C, brown versus orange line, Supplementary Table S2).

The presence of limiting DisA concentrations (1 DisA/100-nt) inhibited (*K*_cat_ of 0.9 ± 0.1 min^−1^), and stoichiometric DisA concentrations (1 DisA/50-nt) blocked (*K*_cat_ of <0.1 min^−1^) RecA nucleation and filament growth (Figure 3C, green and purple line, Supplementary Table S2). This suggests that RecA cannot nucleate onto the quaternary complexes (ssDNA, SsbA, RecO and DisA) and that SsbA and RecO at stoichiometric concentrations were unable to counteract the inhibitory effect of DisA on RecA-mediated ATP hydrolysis.

To test whether DisA impairs RecA filament growth onto ssDNA, RecA was pre-incubated with ssDNA, SsbA and RecO for 5 min, then increasing concentrations of DisA (1 DisA/100- to 25-nt) were added. In the presence of preformed quaternary complexes (ssDNA, RecO, SsbA and RecA), a limiting DisA concentration diminished (*K*_cat_ of 10.1 ± 0.3 min^−1^), and a stoichiometric concentration reduced (*K*_cat_ of 5.0 ± 0.5 min^−1^ at DisA/50-nt) the final steady state rate of ATP hydrolysis (Figure 3D, green and purple line, Supplementary Table S2). Saturating DisA concentration (1 DisA/25-nt) further reduced the ATPase activity of RecA (*K*_cat_ of 2.9 ± 0.2 min^−1^), but it did not block ATP hydrolysis (Figure 3D, red line, Supplementary Table S2). These data altogether suggest that: (i) DisA negatively antagonizes RecA nucleation onto SsbA–ssDNA–RecO complexes, but only partially affects RecA filament growth and (ii) DisA could be responsible for the inhibition of RecA-mediated ATP hydrolysis.

### DisA does not interfere with RecA·dATP filament formation

RecA·dATP has a higher affinity and cooperativity for ssDNA than RecA·ATP (33,34,42), and DisA poorly converts a pair of dATPs into c-di-dAMP (Supplementary Figure S4) (12). To further test whether DisA affects RecA nucleation and filament growth onto ssDNA, we used RecA-mediated dATP hydrolysis as an indirect readout of RecA·dATP binding to ssDNA (31,32), as it was done with ATP. As previously reported (42,53), RecA nucleation and polymerization onto ssDNA showed a biphasic curve with a ∼4 min lag phase, followed by robust dATP hydrolysis, with a maximal rate of 18.1 ± 0.2 min^−1^ (Supplementary Figure S3C, brown line, Supplementary Table S2). DisA pre-incubated with circular ssDNA or linearized dsDNA did not hydrolyse dATP (Supplementary Figure S3C, blue line and insert, blue line).

When DisA was pre-incubated with circular ssDNA, the dATPase activity of RecA was not inhibited (*K*_cat_ of 17.7 ± 0.1 min^−1^) (Supplementary Figure S3C, red and brown lines, Supplementary Table S2). Similarly, when DisA was pre-incubated with ssDNA and linearized dsDNA, and then RecA was added, the lag time and the steady state rate of RecA-mediated dATP hydrolysis were not affected by the presence of DisA (*K*_cat_ of 17.9 ± 0.1 min^−1^) (Supplementary Figure S3C insert, orange and green lines, Supplementary Table S2). It is likely that the hydrolysis of dATP observed in the experiments can solely be attributed to the presence of RecA protein, and DisA barely affected RecA·dATP nucleation and filament growth onto ssDNA. Furthermore, the presence of increasing c-di-AMP concentrations did not affect RecA-mediated dATP hydrolysis (Supplementary Figure S3D).

### DisA suppresses RecA-mediated DNA strand exchange

RecA filament formation requires nucleotide binding, and ATP hydrolysis throughout the filament is required for dissociation of RecA·ADP protomers from ssDNA (31,32). Similarly, *B. subtilis* RecA-mediated DNA strand exchange requires nucleotide binding, and the reaction is more efficient in the presence of ATP hydrolysis (35). Thus, to test if DisA bound to the circular ssDNA [*css*] and/or linear dsDNA [*lds*]) affects RecA·dATP-mediated DNA strand exchange, the three-strand exchange reaction was analysed.

**Figure 4.**
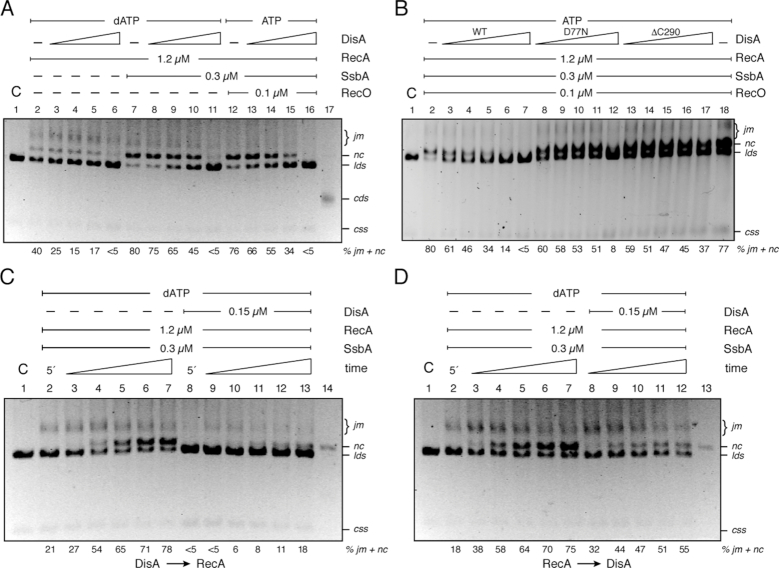
DisA negatively affects recombination. (**A**) Circular 3,199-nt ssDNA (10 μM) and homologous dsDNA (20 μM in nt) were pre-incubated or not with SsbA and then incubated with RecA and increasing DisA concentrations (doubling from 0.025 to 0.2 μM) in buffer B containing 5 mM dATP (lanes 2–11) for 60 min at 37°C. (**B**) The circular ssDNA and homologous linear dsDNA were pre-incubated with SsbA, RecO and then with RecA and increasing DisA (doubling from 0.025 to 0.4 μM), DisA D77N or DisA ΔC290 (doubling from 0.1 to 1.6 μM) concentrations in buffer B containing 5 mM ATP for 60 min at 37°C. (**C**) Circular ssDNA and homologous linear dsDNA were pre-incubated with SsbA and DisA in buffer B containing 5 mM dATP, 5 min later RecA was added, and the reaction incubated for variable time (10, 20, 30, 45 and 60 min) at 37°C. (**D**) Circular ssDNA and homologous linear dsDNA were pre-incubated with SsbA and RecA in buffer B containing 5 mM dATP, then DisA was added and the reaction was incubated for variable time (in min) at 37°C and separated by 0.8% agarose gel electrophoresis. The position of the bands corresponding to substrates (*css, lds*), intermediates (joint molecule, *jm*), products (nicked circular, *nc*) and covalently closed circular duplex DNA (*cds)* are indicated. C denotes the control DNA substrates. The quantification of intermediates/products indicated at the bottom is the mean of ≥3 independent experiments.

RecA·dATP can catalyze DNA strand exchange between *lds* with the complementary *css* substrate in the absence of any accessory protein (33,34,42). RecA·dATP initiated DNA recombination by pairing the *lds* with the complementary *css* substrate, leading to the formation of a joint molecule (*jm*), followed by extensive DNA strand exchange to generate the nicked circular duplex (*nc*) products (Figure 4A, lane 2). The DNA strand exchange was more efficient when SsbA was pre-incubated with the ssDNA, with ∼70% of the substrate converted to *nc* products and ∼10% remaining as *jm* intermediate in 60 min (Figure 4A, lane 7). RecA·ATP, however, requires a two-component mediator (RecO and SsbA) *in vitro* to mediate DNA strand exchange of ∼76% of the substrate (Figure 4A, lane 12) (35). In the absence or the presence of SsbA, RecA·dATP-mediated strand exchange between the *css* and homologous *lds* was reduced 8- to 16-fold by a DisA:RecA molar ratio of 1:6 (Figure 4A, lanes 2 versus 6 and 11). Since DisA does not impair RecA-mediated dATP hydrolysis (Supplementary Figure S3C), it is likely that DisA affects productive RecA-mediated DNA strand exchange.

When dATP was replaced by ATP and the DNA substrates were pre-incubated with the two-component mediator (SsbA and RecO), and then DisA and RecA were added, RecA-mediated DNA strand exchange was inhibited by the addition of increasing DisA concentrations (Figure 4A, lanes 13–16). We can envision that during the search for homology, RecA promotes the formation of *jm*, but accumulation of *nc* products is impaired. DisA itself, its DAC activity or the accumulation of c-di-AMP might affect RecA-mediated productive DNA strand exchange.

To test these hypotheses, DisA was replaced by DisA D77N (a DAC active site mutant), and the three-strand exchange reaction was performed. As revealed in Figure 4B (lane 12), RecA-mediated strand exchange between the *css* and homologous *lds* was reduced ∼10-fold by DisA D77N. To further substantiate whether DisA affects RecA-mediated DNA strand exchange by a direct interaction of the proteins rather than by producing c-di-AMP, the three-strand exchange reaction was performed in the presence of increasing c-di-AMP concentrations. RecA·dATP alone (Supplementary Figure S5, lanes 2 versus 3–7) or RecA·ATP in the presence of SsbA and RecO as mediators (Supplementary Figure S5, lanes 8 versus 9–13) was insensitive to increasing c-di-AMP concentrations. It is likely, therefore, that DisA itself rather than its synthesized product, c-di-AMP, inhibits RecA-mediated DNA strand exchange.

To test whether DisA binds to and competes with RecA for substrate binding, and indirectly inhibits RecA-mediated DNA strand exchange, DisA was replaced by DisA ΔC290 (a mutant lacking the DNA binding domain), and the three-strand exchange reaction was performed. RecA-mediated strand exchange was reduced ∼2-fold by DisA ΔC290, but it did not block the reaction (Figure 4B, lane 17), suggesting that DisA interaction with the DNA substrates is necessary but not sufficient to block RecA-mediated DNA strand exchange.

### DisA interferes with RecA-mediated DNA strand exchange when added before RecA

To gain insights into the inhibition of RecA activity by DisA, the effect of DisA on RecA-mediated DNA strand exchange was assayed by varying the order of protein addition and reaction time course. In the presence of dATP, SsbA and RecA were incubated with *css* and *lds* DNA substrates, and in the first 5 min of reaction generated ∼19% of *jm* (Figure 4C and D, lane 2), while recombinant *nc* products started being formed at 20 min, reaching nearly 65% of *nc* formation at 60 min (Figure 4C and D, lanes 3–7). When DisA was allowed to pre-bind the SsbA–ssDNA complex, RecA-mediated DNA strand exchange is completely blocked. The formation of *nc* products was extremely reduced, rendering less than 20% of *jm* plus *nc* products after 60 min (Figure 4C, lanes 9–13). Interestingly, when RecA-dATP was pre-bound to the SsbA–ssDNA complex, it was able to displace DisA and form *jm* intermediates and *nc* products. The formation of final *nc* products started at 20 min, reaching nearly ∼55% of *jm* plus *nc* products after 60 min (Figure 4D, lanes 8–12). Since RecA was sensitive to order-of-addition effects with DisA, this result indicates that DisA, rather than any indirect effect, down-regulated RecA-mediated DNA strand exchange. DisA bound to the substrate DNA downregulates RecA-mediated strand invasion and pairing *in vitro*, but *in vivo* this delay should provide more time for the repair of DNA lesions, thereby enhancing cell viability.

### RecA does not suppress the rate of c-di-AMP synthesis

We next tested whether RecA-mediated intermediates (as D-loop structures) or RecA protein alone interacts with DisA and blocks c-di-AMP synthesis. To this end, the strand exchange reaction was performed in the presence of [α^32^P]-ATP:ATP (at a ratio of 1:100,000). To measure both the DNA strand exchange and c-di-AMP synthesis they were independently quantified.

**Figure 5.**
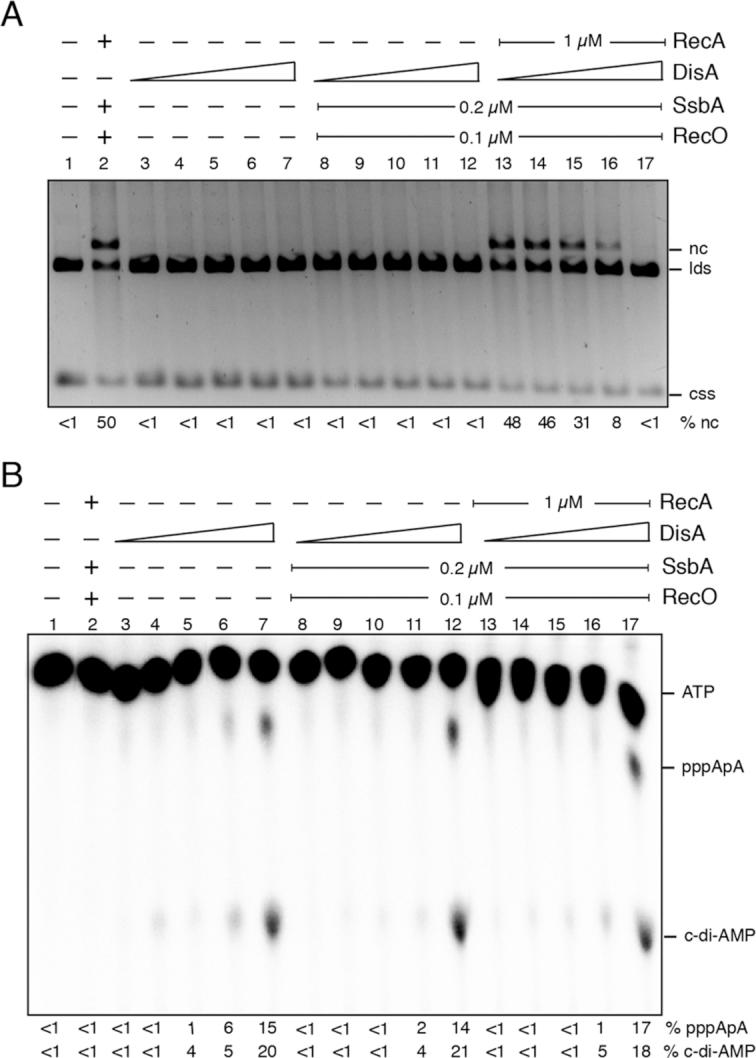
DisA-mediated c-di-AMP synthesis is not affected by RecA·ATP. Circular ssDNA and homologous linear dsDNA were incubated with increasing DisA concentrations (doubling from 0.025 to 0.4 μM) (lanes 3–7), and with SsbA and RecO (lanes 8–12), or SsbA, RecO and RecA (lanes 13–17) in buffer B containing 5 mM ATP and 0.05 μM [α-^32^P]-ATP for 60 min at 37°C. (**A**) The reaction was separated as indicated in Figure 4. In lane 1, the respective *css* and *lds* substrates (termed C) were electrophoresed. The positions of the bands corresponding to *css, lds* and the *nc* products are indicated. The percentage of recombination products (*nc*) is shown. Results are the mean of ≥3 independent experiments. The – denotes the absence of the indicated condition. (**B**) In parallel the DAC activity of DisA was measured. The reaction products were separated by TLC and quantified using ImageJ. The quantification values of relative c-di-AMP amounts are shown, and the positions of the substrate (ATP), the intermediate (pppApA) and the product (c-di-AMP) are indicated.

As revealed in Figure 5A, DisA (lanes 3–7) or DisA, SsbA and RecO (lanes 8–12) did not catalyse DNA strand exchange. In the presence of 0.2 μM DisA only traces of c-di-AMP synthesis were observed, and in the presence of 0.4 μM DisA ∼35% of the 5 mM ATP substrate was converted into pppApA intermediates and c-di-AMP product (Figure 5B, lanes 7 and 12). When the DNA substrates were pre-incubated with increasing DisA and fixed SsbA and RecO concentrations, and then RecA was added, RecA-mediated DNA strand exchange was inhibited at a DisA:RecA molar ratio of 1:5 and blocked at a ratio 1:2.5 (Figure 5A, lanes 13–17). However, pppApA and c-di-AMP synthesis was unaffected (Figure 5B, lanes 13–17). This is consistent with the observation that DisA interacts with and inhibits RecA-mediated DNA strand exchange rather than RecA inhibits the DAC activity of DisA. We consider unlikely that the order of protein addition affects DisA assembly, because DisA inhibits RecA-mediated ATP hydrolysis and DNA strand exchange rather than stimulates the efficiency of the reaction.

### DisA suppresses RecA-mediated DNA strand exchange in the absence of ATP hydrolysis

DisA can convert two molecules of the poorly hydrolysable ATP analogue (ATPγS) into c-di-AMP (15), and RecA·ATPγS can catalyze DNA strand exchange only in the presence of SsbA and RecO, albeit with ∼2-fold lower efficiency than RecA·ATP (Supplementary Figure S6, lanes 2 vs 12) (35). To test whether DisA, by inhibiting ATP hydrolysis-dependent RecA-DNA binding dynamics, or by binding to the recombination products produced by RecA, prevents DNA strand exchange, and if c-di-AMP synthesized *in cis* exerted a negative effect in RecA-mediated DNA strand exchange, the reaction was performed in the presence of ATPγS (Supplementary Figure S6). When ATP was replaced by ATPγS, RecA-mediated DNA strand exchange was ∼18-fold reduced by a DisA:RecA molar ratio of 1:48 (Supplementary Figure S6, lanes 2 versus 3), and the formation of final *nc* products was inhibited in the presence of higher DisA concentrations (DisA:RecA molar ratio of 1:6) (Supplementary Figure S6, lane 6). In contrast, when RecA·ATPγS was added before DisA, the formation of final *nc* products was not affected after 60 min (Supplementary Figure S6, lanes 2 vs 11). It is likely that: (i) DisA bound to RecA and to the recombination product inhibits RecA-mediated DNA strand exchange even in the absence of binding dynamics and (ii) c-di-AMP synthesised *in cis* does not contribute to the inhibition of RecA-mediated DNA strand exchange.

## DISCUSSION

Here we report that during unperturbed spore morphogenesis *B. subtilis* DisA forms a focus that dynamically moves in Δ*addAB* Δ*recJ*, Δ*recO*, Δ*recA* or in the *wt* context. In response to DNA damage, DisA pauses in *wt* and in Δ*addAB* Δ*recJ* cells, but it maintains its dynamic movement during spore morphogenesis in Δ*recO* or Δ*recA* cells. Thus, the signal(s) sensed and recognized by DisA is independent of both AddAB- and RecJ-mediated end resection and of SsbA-coated ssDNA regions. The signal(s) is formed at or downstream of RecO and RecA engagement with ssDNA regions. RecO protein has two activities: to recruit RecA onto SsbA-coated ssDNA and to mediate annealing of complementary ssDNAs coated by the SsbA protein (31,32,54). Since DisA maintains its dynamic movement in Δ*recO* or Δ*recA* cells in the presence of DNA damage, we favour the first activity of RecO that is essential for RecA-mediated DNA strand exchange (35). Not all bacteria, however, respond to DNA damage in a similar way, for example γ-Proteobacteria (best represented by *E. coli* cells) lack DisA. Based on our biochemistry data, we propose that DisA physically interacts with RecA on sites of DNA damage to inhibit its activities. We show that DisA interacts with and targets a RecA nucleoprotein filament and/or RecA-mediated recombination intermediate (D-loops, stalled or reversed replication forks). This is consistent with the observation that: i) DisA neither impairs nor stimulates PriA-dependent initiation of DNA replication *in vitro* (13); ii) the *disA* gene is epistatic with *recA* in response to replicative stress during spore revival or during vegetative growth (12,13), and iii) DisA, RecO and RecA are crucial for survival of DNA-damaged inert mature *B. subtilis* haploid spores revived in unperturbed conditions, and where DSB repair by homologous recombination is not operative (13,24).

Collectively, the study presented here emphasizes the importance of understanding how DisA contributes to overcome a replicative stress in exponentially growing cells (12). Previously, it has been shown that DisA contributes to the two distinct modes of DNA damage tolerance (error-prone and error-free). DisA modulates the contribution of translesion synthesis polymerases, which catalyze stable, although often erroneous, nucleotide incorporation opposite to damaged template bases in *B. subtilis* cells to allow DNA synthesis to resume (13). In unperturbed exponential growth, DisA, which pauses in genetic backgrounds that accumulate a significant amount of unresolved HJs and stalled forks (as in the absence of a branch migration translocase or a HJ resolvase), might modulate replication fork reversal to circumvent such replicative arrest (11,12). DisA, in concert with RecA, is crucial to circumvent damaged template bases during the revival of *B. subtilis* haploid spores, which lack an intact homologous template, via error-free template switching (13,24).

One of the possible signals recognized by DisA is the RecA nucleoprotein filament. The chaperone activity of a RecA nucleoprotein filament promotes the transcriptional repressor, LexA, to cleave itself (55,56), thereby de-repressing ∼40 genes involved in the SOS response, being *recA* among them (31,57). If DisA alters the stability or turnover of the RecA nucleoprotein filaments, in the absence of DisA the level of RecA expression should be altered. Upon DNA damage the increase in RecA expression was indistinguishable between *wt* and *disA* cells (12), suggesting that the formation of a RecA nucleoprotein filament is not the DisA target *in vivo*.

*In vitro*, a RecA nucleoprotein filament invades a homologous dsDNA molecule, resulting in a D-loop intermediate, and RecA binding to a stalled fork catalyses its reversal, resulting in a HJ-like structure (18,20–22,28). DisA-bound to D-loop and HJ DNA suppresses its DAC activity (12,15), suggesting that DisA and RecA interact with those recombination intermediates, and the former mediates checkpoint activation by coordinating the relative timing of molecular events during DNA repair. Indeed, RecA must be tightly regulated to ensure that recombination does not occur at inappropriate times or places in the genome, and when it occurs the recombination event must be correctly coordinated with DNA synthesis (25,31).

We show that DisA inhibits RecA-mediated DNA pairing and *jm* formation by occupying newly exposed ssDNA and also by a protein-protein interaction, as highlighted by DisA ΔC290 inhibition of RecA-mediated ATP hydrolysis (Figure 3A and B). The presence of the two-component mediator, SsbA and RecO, is not sufficient to antagonise the inhibitory effect of DisA. The competitive nature of DisA and RecA·ATP in ssDNA binding is illustrated by the order of addition effect on RecA-mediated ATP hydrolysis (Figure 3C and D). In contrast, DisA cannot affect RecA·dATP nucleation and polymerization onto ssDNA or SsbA-coated ssDNA, suggesting that RecA·dATP could outcompete DisA for binding to ssDNA. We do not consider such assumption relevant because the presence of DisA inhibits both RecA·dATP- and RecA·ATP-mediated DNA strand exchange, however this activity requires nucleotide binding but not its hydrolysis. We assumed that DisA interacts with a RecA filament on the ssDNA and on the *jm* intermediate and stabilizes RecA on the heteroduplex DNA, thus attenuates the DNA strand exchange reaction. We suspect that the attenuation gave specific repair enzymes sufficient time to remove the lesion. Such delay, however, is not observed if DisA was added after RecA.

DisA and its DAC active site mutant (DisA D77N) inhibit the ATPase and DNA strand exchange activities of RecA, suggesting that DisA rather than its product, c-di-AMP, is responsible for such inhibition. Indeed, addition of a large excess of c-di-AMP neither affects the ATPase nor the DNA strand exchange RecA activities. It is assumed that DisA interacts with and suppresses the RecA ATPase activity, thus stabilizing RecA on the DNA. Since DisA ΔC290 impairs RecA·ATP nucleation and filament growth, but marginally inhibits RecA-mediated DNA strand exchange, we consider unlikely that DisA exerts its inhibitory effect solely by inhibiting ATP hydrolysis-dependent DNA binding dynamics. This is consistent with the observation that DisA suppressed RecA·ATPγS-mediated DNA strand exchange and this effect was also dependent on the order of DisA addition, suggesting that both inhibition of RecA assembly/disassembly and binding to the recombination intermediates downregulate RecA activities.

**Figure 6.**
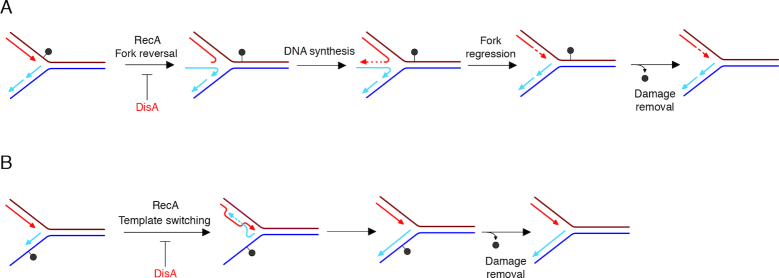
Proposed mechanisms for DisA in RecA-mediated DNA damage tolerance pathways. (**A**) An unrepaired DNA lesion on the leading strand template (grey dot) causes blockage of replication fork movement. DisA regulates RecA-mediated annealing of the nascent strands, reversal of the leading and lagging daughter strands to form a HJ DNA structure. Then, DNA synthesis of the DNA complementary to the damaged site (denoted by dotted line) is followed by fork regression. (**B**) An unrepaired DNA lesion on the lagging strand template (grey dot) causes blockage of replication fork movement. DisA regulates RecA-mediated annealing of the nascent strands with DNA synthesis initiating from the alternative template (template switching). Removal of the lesion by a specific repair pathway then occurs.

We propose that DisA binds to its cognate signal, a stalled or reversed fork (Figure 6A–B). Then, DisA might downregulate RecA-mediated pairing of the strand of the sister duplex by fork reversal to overcome leading strand blocks (Figure 6A), or RecA-mediated invasion of the opposite homologous DNA duplex by the stalled nascent strand, and subsequent DNA synthesis using as a template the undamaged strand to overcome such discontinuities (template switching) (Figure 6B). The competitive nature of DisA variants in RecA·ATP nucleoprotein filament formation is illustrated by the order of addition effect on DNA strand exchange reactions (Figure 4). In other words, DisA might provide the timing to facilitate the removal of the DNA lesion. After damage removal, the levels of c-di-AMP increase, DNA synthesis restart and growth-related processes resume. Alternatively, in a fraction of cells, RecA binds first to a stalled or reversed fork and becomes insensitive to DisA action. The DNA lesions are overcome by RecA-mediated fork reversal or template switching without delay. These two conditions, DisA or RecA first, generate two sub-populations to increase the survival under certain growth condition (e.g. sporulating and exponentially growing cells). The molecular bases of DisA regulation of RecA activities might be conserved, but the molecular bases of the regulatory mechanism may be different among distantly related bacteria. For example, in *M. smegmatis*, c-di-AMP interacts with RecA and attenuates DNA strand exchange through the disassembly of the RecA nucleoprotein filaments (58).

## Supplementary Material

gkz219_Supplemental_Files

## Notes

Present address: Carolina Gándara, Institute of Neuroscience, Faculty of Medical Sciences, Newcastle University, NE24HH, Newcastle-upon-Tyne, UK.
