# Supplementary material for: Bacillus subtilis DisA regulates RecA-mediated DNA strand exchange
Source: Nucleic Acids Res. 2019 Mar 27;47(10):5141–54. doi: 10.1093/nar/gkz219 (PMC6547438; doi:10.1093/nar/gkz219)
Supplement: gkz219_Supplemental_Files [file gkz219_supplemental_files.zip › Torres et al Supplemental.pdf]

Bacillus subtilis DisA regulates RecA-mediated DNA strand exchange

Rubén Torres[1,†], Begoña Carrasco[1,†], Carolina Gándara[1,*], Amit K. Baidya[2], Sigal Ben-Yehuda[2,§] and Juan C. Alonso[1,§]

Department of Microbial Biotechnology, Centro Nacional de Biotecnología, CNB-CSIC, 3 Darwin, 28049 Madrid, Spain.

Annex 1. **DisA ΔC290 impairs cells viability**

The wild-type (*wt*) and *disA* ΔC290 or *disA* D77N mutant genes under the control of an IPTG inducible promoter were ectopically integrated into the *amyE* locus of competent *B. subtilis* Δ*disA* cells to avoid any negative effect on the downstream stress response *yacL* gene, which could potentially occur if integrated into the native locus. In the generated Δ*disA disA*$^+$, Δ*disA disA* ΔC290 and Δ*disA disA* D77N mutant strains (see Table S1), the only *disA* gene expressed in the background was the ectopically integrated *wt* or mutant variant; thus, for simplicity, we refer to them as *disA*$^+$, *disA* ΔC290 and *disA* D77N strains. The repair-by-recombination ability of the exponentially growing *disA*$^+$, *disA* ΔC290 or *disA* D77N mutant strains was tested by chronically exposing the cells to methyl methanesulfonate (MMS) or 4-nitroquinoline-1-oxide (4NQO), and its survival was then compared with the one of *rec*$^+$ (*disA* gene expressed from its native locus and promoters) cells.

We found that *disA* ΔC290 or *disA* D77N gene from an ectopic locus (*amyE*) did not affect cell viability in the absence of the IPTG induction in Δ*disA* or *rec*$^+$. Unexpectedly, the viability of *disA* ΔC290 cells was significantly reduced upon IPTG addition (1 mM) (Fig. 2D). A *disA* ΔC290 mutation showed a significant reduction in MMS or 4NQO survival, indicating that the allele produces dominant-negative phenotypic effects when compared to Δ*disA* or *disA* D77N cells (Fig. S2D). A *disA* ΔC290 mutation also interfered the wild type protein function, because the allele produces a dominant-negative phenotypic effect and interferes *rec*$^+$ cells (Fig. S2D).

Annex 2. **DisA interacts with RecA**

To test whether DisA interacts with RecA, their coding region was fused to either the T18 or T25 domain of *Bordetella* adenylate cyclase. If the fused pair interact, it should reconstitute the adenylate cyclase to generate cAMP, which then activates the cAMP-bound catabolite activator protein (CAP) to induce the expression of ß-galactosidase (Fig. 2A). Using a bacterial two-hybrid assay we confirmed that full-length RecA and DisA interact with itself and with each other by qualitatively assessing the appearance of blue color in the colony due to the breakdown of X-gal in the medium (Fig. 2B).

To confirm this interaction *in vitro*, His-tagged DisA was bound to a Ni^{2+} agarose column through coordination with the C-terminal histidine tag, in the absence or presence of ssDNA and ATP·Mg^{2+}, and the retention of RecA in the Ni^{2+} matrix analyzed (Fig. 2C-D). DisA (pI 5.7) and RecA (pI 5.0), in the ATP bound form, bind ssDNA cooperatively and form nucleoprotein complexes and filaments, respectively (1-4). Purified His-tagged DisA (predicted mass 41.6 kDa) showed two discrete bands of 41 and 42 kDa (see Materials and methods), that correlate with fast moving apo DisA and slow-moving c-di-AMP-bound DisA, respectively (Fig. 2C, lane 1) (3,4). His-tagged apo DisA was mainly retained on the 50-µl Ni^{2+} column, although a fraction was detected in the flow-through (FT) (Fig. 2C, lane 3). When DisA was eluted (E) with Buffer A containing 1 M NaCl and 0.4 M imidazole, only apo DisA was detected (Fig. 2C, lane 5). RecA

(predicted mass 38.0 kDa, and migrating with an expected mass of 41.5 kDa) was partially entrapped in the Ni^{2+} microcolumn as judged by the flow-through fraction (Fig. 2C, lane 6). After extensive washing, no RecA protein was detected upon elution with Buffer A containing 1 M NaCl and 0.4 M imidazole (Fig. 2C, lane 8). When both DisA and RecA were loaded into the Ni^{2+} column, we observed that a fraction of DisA and RecA were present in the flow-through (FT) and in a minor extent in the washes (data not shown), both in the presence or absence of ssDNA and ATP (Fig. 2D, lanes 3-4 and 6-7). It is likely that RecA free in solution could destabilize DisA bound to the Ni^{2+} matrix with a stoichiometry of ~1 (Fig. 2D, lanes 3 and 6). DisA and a reduced fraction of RecA co-eluted from the matrix during the elution step in the presence or the absence of ssDNA and ATP (Fig. 2D, lanes 5 and 8). A Western blot of these experiments, with anti-RecA polyclonal antibody or anti-His monoclonal antibodies, revealed specific signals at the positions of RecA and DisA (Fig. 2D, lanes 5 and 8), and suggested that DisA transiently interacts with RecA both in the presence or absence of ssDNA and ATP.

Annex 3. **DisA did not shrink the nucleotide pool at optimal conditions for RecA**
DisA preferentially converts a pair of ATPs into c-di-AMP when compared to dATP (or dADP) (3); whereas RecA in the dATP form preferentially nucleates and polymerizes onto ssDNA when compared to RecA bound to ATP (5,6). RecA nucleation onto ssDNA and DisA DAC activity are optimal at 10 mM Mg^{2+} (3,7), thus 10 mM magnesium acetate concentrations were used for any further analyses. To test whether DisA activity is affected under the experimental conditions used for RecA assays, we purified DisA and RecA proteins and the synthesis of c-di-(d)AMP in the presence of increasing concentrations of ATP or dATP was measured.

At about its K_m for ATP (0.1 mM), DisA converted [α^{32}P]-ATP:ATP into c-di-AMP with a catalytic rate constant (K_{cat}) of ~70 min^{-1}, and under this condition ~80% of ATP was converted into c-di-AMP in a 30 min reaction (Fig. S4) (4). The DAC activity of DisA occurred in the presence of 10 mM Mg^{2+} and to a lesser extent in presence of Mn^{2+} (data not shown). The DAC activity of DisA increased with increasing amounts of ATP up to ~0.3 mM, and decreased at concentrations higher than ~0.8 mM, to be strongly reduced at 5 mM ATP (Fig. S3), suggesting that DisA did not obey Michaelis-Menten kinetics under our experimental conditions. When ATP was replaced by dATP, DisA was unable to convert two dATP molecules into c-di-dAMP at any of the dATP concentrations tested (Fig. S3). Previously, an 8-fold excess of DisA is required to hydrolyse ~10% of the 0.1 mM dATP, with the accumulation of the dATP-dAMP intermediate and traces of c-di-dAMP (4).

These data altogether suggest that under the experimental conditions used to measure RecA-mediated nucleotide hydrolysis (5 mM [d]ATP and 10 mM magnesium acetate), the ATP or dATP pools were marginally reduced or unaffected, while the activity of DisA is preserved (Fig. S3).

References

1. Cox, M.M. (2007) Regulation of bacterial RecA protein function. *Crit Rev Biochem Mol Biol*, **42**, 41-63.
2. Bell, J.C. and Kowalczykowski, S.C. (2016) RecA: Regulation and Mechanism of a Molecular Search Engine. *Trends Biochem Sci*, **41**, 491-507.
3. Witte, G., Hartung, S., Buttner, K. and Hopfner, K.P. (2008) Structural biochemistry of a bacterial checkpoint protein reveals diadenylate cyclase activity regulated by DNA recombination intermediates. *Mol Cell*, **30**, 167-178.

4. Gándara, C., de Lucena, D.K.C., Torres, R., Serrano, E., Altenburger, S., Graumann, P.L. and Alonso, J.C. (2017) Activity and *in vivo* dynamics of *Bacillus subtilis* DisA are affected by RadA/Sms and by Holliday junction-processing proteins. *DNA Repair (Amst)*, **55**, 17-30.
5. Yadav, T., Carrasco, B., Myers, A.R., George, N.P., Keck, J.L. and Alonso, J.C. (2012) Genetic recombination in *Bacillus subtilis*: a division of labor between two single-strand DNA-binding proteins. *Nucleic Acids Res*, **40**, 5546-5559.
6. Lovett, C.M., Jr. and Roberts, J.W. (1985) Purification of a RecA protein analogue from *Bacillus subtilis*. *J Biol Chem*, **260**, 3305-3313.
7. Carrasco, B., Manfredi, C., Ayora, S. and Alonso, J.C. (2008) *Bacillus subtilis* SsbA and dATP regulate RecA nucleation onto single-stranded DNA. *DNA Repair (Amst)*, **7**, 990-996.
8. Ceglowski, P., Luder, G. and Alonso, J.C. (1990) Genetic analysis of *recE* activities in *Bacillus subtilis*. *Mol Gen Genet*, **222**, 441-445.
9. Sanchez, H., Kidane, D., Cozar, M.C., Graumann, P.L. and Alonso, J.C. (2006) Recruitment of *Bacillus subtilis* RecN to DNA double-strand breaks in the absence of DNA end processing. *J Bacteriol*, **188**, 353-360.
10. Fernández, S., Kobayashi, Y., Ogasawara, N. and Alonso, J.C. (1999) Analysis of the *Bacillus subtilis recO* gene: RecO forms part of the RecFLOR function. *Mol Gen Genet*, **261**, 567-573.
11. Gándara, C. and Alonso, J.C. (2015) DisA and c-di-AMP act at the intersection between DNA-damage response and stress homeostasis in exponentially growing *Bacillus subtilis* cells. *DNA Repair (Amst)*, **27**, 1-8.
12. Cañas, C., Carrasco, B., Ayora, S. and Alonso, J.C. (2008) The RecU Holliday junction resolvase acts at early stages of homologous recombination. *Nucleic Acids Res*, **36**, 5242-5249.
13. Manfredi, C., Carrasco, B., Ayora, S. and Alonso, J.C. (2008) *Bacillus subtilis* RecO nucleates RecA onto SsbA-coated single-stranded DNA. *J Biol Chem*, **283**, 24837-24847.

Table S1. *Bacillus subtilis* strains used

Strain	Relevant genotype[a]	Source
BG214	*rec*$^{+}$	Lab. strain
BG1733	+ *disA-gfp:spc*	This work
BG190	+ Δ*recA*	(8)
BG1737	+ Δ*recA disA-gfp:spc*	This work
BG1429	+ Δ*addAB* Δ*recJ*	(9)
BG1743	+ Δ*addAB* Δ*recJ disA-gfp:spc*	This work
BG439	+ Δ*recO*	(10)
BG1741	+ Δ*recO disA-gfp:spc*	This work
BG1745	+ *disA* ΔC290-*gfp:kan*	This work
BG1791	+ *amy:spc*:*disA* ΔC290	This work
BG1221	+ Δdis*A*	(11)
BG1793	+ Δdis*A amy:spc*:*disA*	This work
BG1797	+ Δdis*A amy:spc*:*disA* ΔC290	This work
BG1795	+ Δdis*A amy:spc*:*disA* D77N	This work

[a]All strains are derivatives of *B. subtilis* BG214 (*trpCE metA*5 *amyE1 ytsJ*1 *rsbV*37 *xre*1 *xkd*A1 *att*^{SPß} *att*$^{\text{ICE}Bs1}$).

Table S2. Rates of ssDNA-dependent (d)ATP hydrolysis and lag time measurements

Proteins[a]	Condition	Lag time[a] (in min)	k_{cat} min^{-1}[a]
RecA (1 RecA/ 12-nt)	dATP[b]<br>ATP[b]	4 ± 0.5<br>< 1	18.1 ± 0.2<br>8.9 ± 0.3
DisA (1 DisA/ 50-nt)	dATP<br>ATP	NA<br>NA	<0.1<br><0.1
(DisA +ssDNA) + RecA	dATP<br>ATP	4 ± 0.6<br>NA	17.7 ± 0.1<br>0.8 ± 0.2
(DisA+ ssDNA + dsDNA) + RecA	dATP<br>ATP	5 ± 0.3<br>ND	17.9 ± 0.1<br>ND
(RecO + SsbA + ssDNA) +RecA<br>(RecO + SsbA + ssDNA) +RecA +DisA[c]<br>(RecO + SsbA + ssDNA) +RecA +DisA[d]	ATP<br>ATP<br>ATP	5 ± 0.3<br>NA<br>NA	18.0 ± 0.3<br>0.9 ± 0.1<br><0.2
(RecO + SsbA + ssDNA +RecA) +DisA[c]<br>(RecO + SsbA + ssDNA +RecA) +DisA[d]<br>(RecO + SsbA + ssDNA +RecA) +DisA[e]	ATP<br>ATP<br>ATP	5 ± 0.1<br>2 ± 0.2<br>1 ± 0.3	10.1 ± 0.3<br>5.0 ± 0.5<br>2.9 ± 0.2

[a]Rates of RecA-mediated (d)ATP hydrolysis and the nucleation lag times were measured as indicated in materials and methods. [b]RecA-mediated (d)ATP hydrolysis and the lag time were reported elsewhere (5,12,13), and determined here again for a direct comparison. The protein(s) pre-incubated with ssDNA are denoted between parentheses. The stoichiometry of monomeric DisA is indicated (DisA/100[c]-, 50[d]- or 25[e]-nt). The steady state kinetic parameters for RecA (1 RecA/ 12-nt) were derived from the data presented in Fig. 3 and S3. The average rate of (d)ATP hydrolysis were obtained from more than three independent experiments. NA, not applied; ND, not determined.

Figure Legend

Fig. S1. DisA forms static foci upon DNA damage in *wt* cells. (A–B) Dynamic localization of DisA-GFP foci demonstrated by time-lapse microscopy from individual cells of the DisA-GFP-producing strain. Time-lapse microscopy images (400 ms intervals) from DisA-GFP-producing strains stained with DAPI (blue), FM 4-64 (red) and DisA-GFP (green) seeing as foci in unperturbed *wt* cells (A). The corresponding movie is displayed in the Supplemental data (Movie S1). Time-lapse microscopy images (400 ms intervals) from DisA-GFP-producing strains in *wt* cells (B) upon addition of 350 µg/ml NAL at the onset of sporulation. The corresponding movies are displayed in the Supplemental data (Movie S2). Selected images were chosen for demonstration. Scale bars correspond to 5 µm.

Fig. S2. The DNA binding domain is required for DisA focus motion on DNA. (A–B) Dynamic localization of DisA ΔC290-GFP foci demonstrated by time-lapse microscopy from individual cells of the DisA ΔC290-GFP-producing strain. Time-lapse microscopy images (400 ms intervals) from DisA ΔC290-GFP-producing strains stained with DAPI (blue), FM 4-64 (red) and DisA ΔC290-GFP (green) seeing as foci in unperturbed *wt* cells (A and C) and upon addition of 350 µg/ml NAL at the onset of sporulation (B and D). The corresponding movies are displayed in the Supplemental data (Movies S9 and 10, respectively). Selected images were chosen for demonstration. Scale bars correspond to 5 µm. (E) DAC activity of DisA mutant variants. DisA, DisA ΔC290 or DisA D77N (0.1-1.6 µM) was incubated in buffer B containing 0.1 mM ATP and 0.05 µM [α-^{32}P]-ATP for 30 min at 37 °C. The reaction products were separated by TLC and quantified using ImageJ. The quantification values of relative c-di-AMP amounts are shown. (F) Survival of strains expressing DisA mutant variants, from an IPTG inducible promoter, grown to middle exponential phase upon chronic exposure to MMS and 4NQO. The *wt* (BG214), Δ*disA* (BG1221), Δ*disA disA*$^{+}$ (BG1793), Δ*disA disA* ΔC290 (BG1797), Δ*disA disA* D77N (BG1795) and *wt disA* ΔC290 (BG1791) cells were grown to reach exponential phase (OD_{560}= 0.4) in LB medium at 37ºC, serially diluted, and 10 µl of serial 10-fold dilutions (10^{-3} to 10^{-6}) were spotted on LB plates containing the indicated concentration of MMS or 4NQQO and IPTG, or in the absence of the drug (-) plus/minus IPTG. Plates were incubated overnight at 37ºC. The results are representative of at least four independent experiments.

Fig. S3. RecA-mediated (d)ATPase activity. (A) Circular 3.199-nt ssDNA (10 µM in nt) was incubated with RadA/Sms (0.4 µM), DisA (0.4 µM) or both in buffer B containing 5 mM ATP and the ATPase activity measured for 30 min. (B) Circular ssDNA was incubated with RecA (0.8 µM) and increasing c-di-AMP concentrations (0.1, 1 and 5 mM) in buffer B containing 5 mM ATP, and ATPase activity was measured for 30 min. Insert, Circular ssDNA and linear dsDNA were incubated with RecA, DisA or both in buffer B containing 5 mM dATP, and dATPase activity was measured for 30 min. (C) Circular ssDNA was incubated with RecA in buffer B containing 5 mM dATP and dATPase activity was measured for 30 min. (D) Circular 3.199-nt ssDNA (10 µM in nt) was incubated with RecA (0.8 µM) and increasing c-di-AMP concentrations (0.1, 1 and 5 mM) in buffer B containing 5 mM dATP, and dATPase activity was measured for 30 min. All reactions were repeated three or more times with similar results.

Fig. S4. DAC activity of DisA in the presence of increasing concentrations of the nucleotide cofactor. DisA (0.2 µM) was incubated in buffer B containing 0.1, 1 and 5 mM (d)ATP and 0.05 µM [α-^{32}P]-ATP for 40 min at 37 °C. The reaction products were separated by TLC and quantified

using ImageJ. The quantification values of relative c-di-AMP amounts are shown.

Fig. S5. RecA·ATP-mediated DNA strand exchange in the presence of increasing c-di-AMP concentrations. Circular ssDNA and homologous linear dsDNA were incubated with RecA and increasing c-di-AMP concentrations (doubling from 0.3 to 5 mM) in buffer B containing 5 mM dATP (lanes 2-7) for 60 min at 37 °C. The circular ssDNA and homologous linear dsDNA were pre-incubated with SsbA, RecO, RecA and increasing c-di-AMP concentrations in buffer B containing 5 mM ATP (lanes 8-13) for 60 min at 37 °C. The reaction was separated as indicated in Fig. 4. In lane 1, the respective *css* and *lds* substrates (termed C) were electrophoresed. The positions of the bands corresponding to *css*, *lds*, *jm* and *nc* products are indicated. The percentage of intermediates (*jm)* plus recombination products (*nc)* are shown. Results are the mean (± SEM) of ≥3 independent experiments. The - denotes the absence of the indicated condition.

Fig. S6. DisA negatively affects RecA·ATPγS-mediated recombination. Circular ssDNA and homologous linear dsDNA were pre-incubated with SsbA, RecO and increasing DisA concentrations (0.025 to 0.2 µM) in buffer B containing 5 mM ATPγS, then RecA was added and the reaction incubated for 60 min at 37 °C (lanes 2-6). Circular ssDNA and homologous linear dsDNA were pre-incubated with SsbA, RecO and RecA in buffer B containing 5 mM ATPγS, then increasing DisA concentrations were added and the reaction incubated for 60 min at 37 °C (lanes 7-11). The reaction was separated as indicated in Fig. 4. In lane 1, the respective *css* and *lds* substrates (termed C) were electrophoresed. The positions of the bands corresponding to *css*, *lds*, *jm* and *nc* products are indicated. The percentage of recombination intermediates (*jm*, grey bars) and products (*nc*, open bars) are shown. Results are the mean (± SEM) of ≥3 independent experiments. The - denotes the absence of the indicated condition.

Figure S1

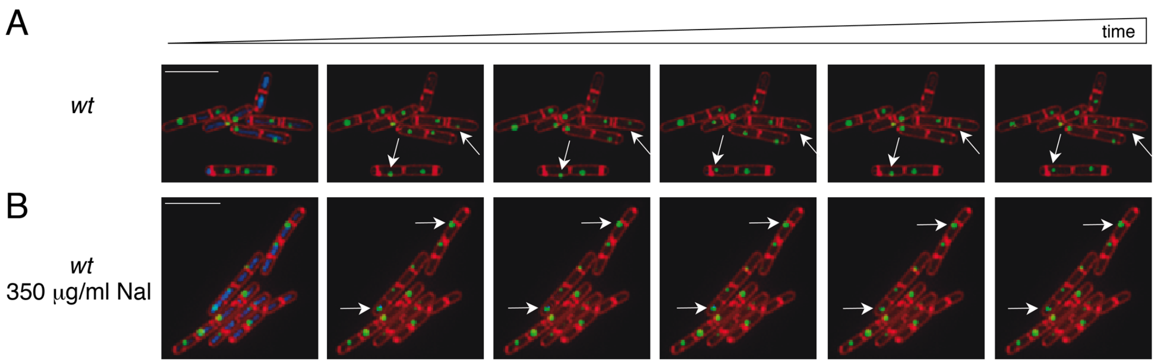

Figure S2

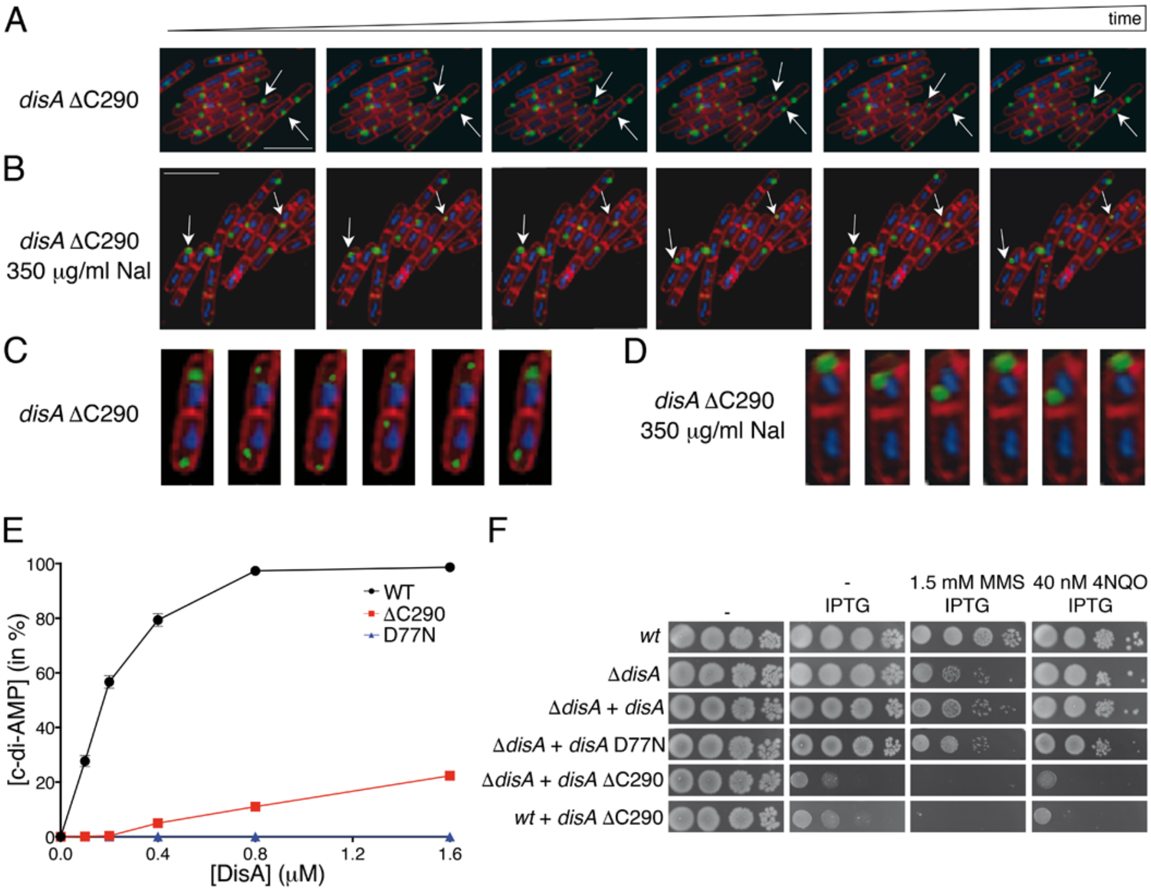
A
time
disA ΔC290
B
disA ΔC290
350 μg/ml Nal
C
disA ΔC290
D
disA ΔC290
350 μg/ml Nal
E
[c-di-AMP] (in %)
100
80
60
40
20
0
0.0
0.4
0.8
1.2
1.6
[DisA] (μM)
WT
ΔC290
D77N
F
-
-
IPTG
1.5 mM MMS
IPTG
40 nM 4NQO
IPTG
wt
ΔdisA
ΔdisA + disA
ΔdisA + disA D77N
ΔdisA + disA ΔC290
wt + disA ΔC290

Figure S3

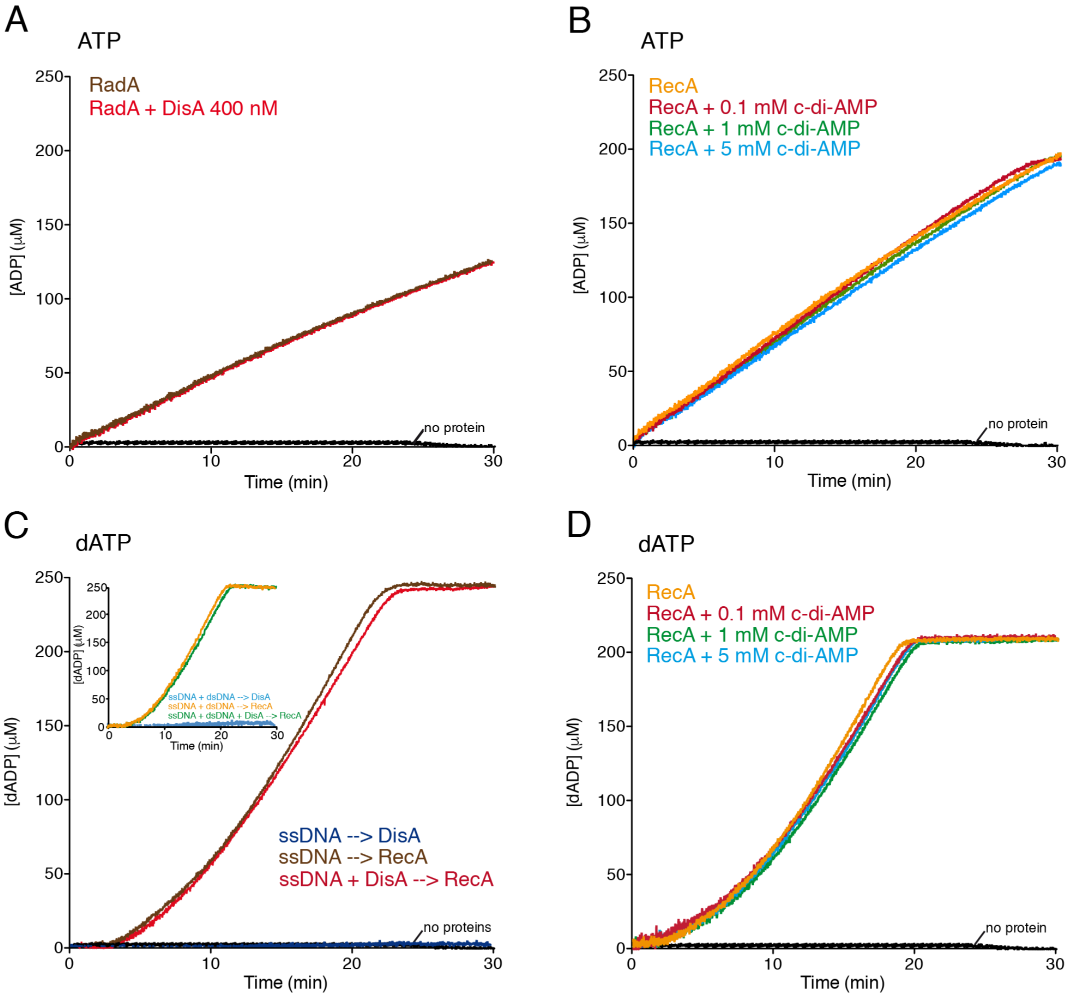
A
ATP
RadA
RadA + DisA 400 nM
[ADP] (μM)
no protein
Time (min)
B
ATP
RecA
RecA + 0.1 mM c-di-AMP
RecA + 1 mM c-di-AMP
RecA + 5 mM c-di-AMP
[ADP] (μM)
no protein
Time (min)
C
dATP
[dADP] (μM)
ssDNA + dsDNA --> DisA
ssDNA + dsDNA --> RecA
ssDNA + dsDNA + DisA --> RecA
Time (min)
ssDNA --> DisA
ssDNA --> RecA
ssDNA + DisA --> RecA
no proteins
Time (min)
D
dATP
RecA
RecA + 0.1 mM c-di-AMP
RecA + 1 mM c-di-AMP
RecA + 5 mM c-di-AMP
[dADP] (μM)
no protein
Time (min)

Figure S4

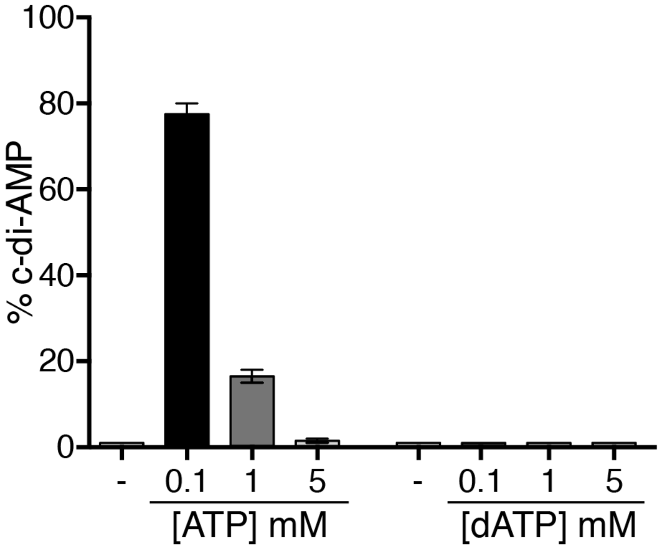

Figure S5

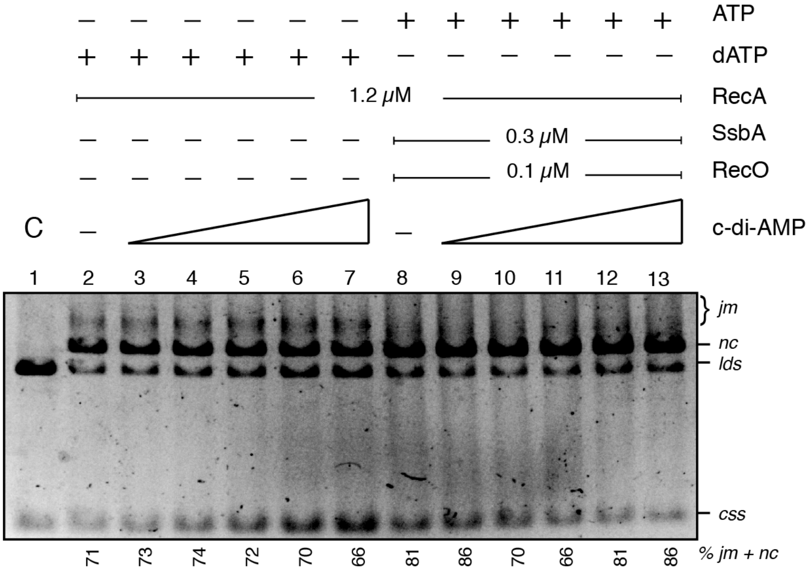

C
ATP
dATP
RecA
1.2 μM
SsbA
0.3 μM
RecO
0.1 μM
c-di-AMP
1 2 3 4 5 6 7 8 9 10 11 12 13
jm
nc
lds
css
71 73 74 72 70 66 81 86 70 66 81 86
% jm + nc

Figure S6

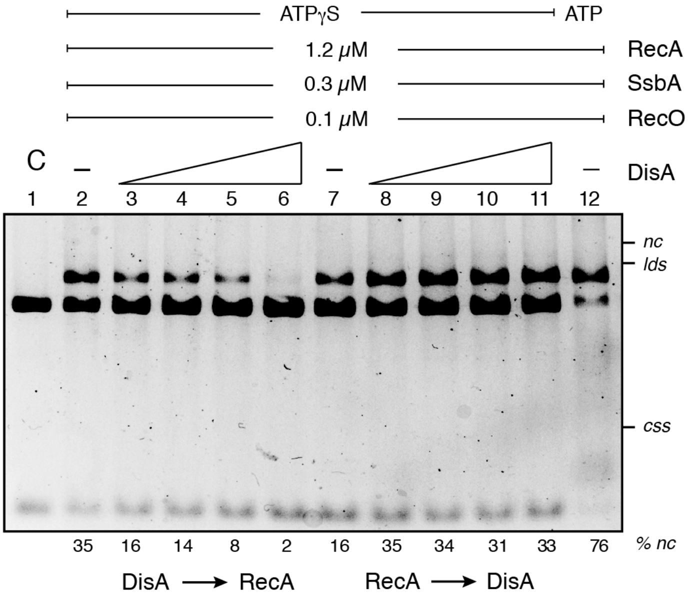
ATPγS
ATP
1.2 μM
RecA
0.3 μM
SsbA
0.1 μM
RecO
C
DisA
1 2 3 4 5 6 7 8 9 10 11 12
nc
lds
css
35 16 14 8 2 16 35 34 31 33 76
% nc
DisA → RecA
RecA → DisA
